# SIRPα engagement regulates ILC2 effector function and alleviates airway hyperreactivity via modulating energy metabolism

**DOI:** 10.1038/s41423-024-01208-z

**Published:** 2024-08-19

**Authors:** Yoshihiro Sakano, Kei Sakano, Benjamin P. Hurrell, Pedram Shafiei-Jahani, Mohammad Hossein Kazemi, Xin Li, Stephen Shen, Richard Barbers, Omid Akbari

**Affiliations:** 1 Department of Molecular Microbiology and Immunology, Keck School of Medicine, University of Southern California, Los Angeles, CA USA; 2 Department of Clinical Medicine, Division of Pulmonary and Critical Care Medicine, Keck School of Medicine of USC, University of Southern California Hospital, Los Angeles, CA USA

**Keywords:** SIRPa, CD47, ILC2, AHR, Asthma, Innate lymphoid cells, Asthma

## Abstract

Group-2 innate lymphoid cells (ILC2) are part of a growing family of innate lymphocytes known for their crucial role in both the development and exacerbation of allergic asthma. The activation and function of ILC2s are regulated by various activating and inhibitory molecules, with their balance determining the severity of allergic responses. In this study, we aim to elucidate the critical role of the suppressor molecule signal regulatory protein alpha (SIRPα), which interacts with CD47, in controlling ILC2-mediated airway hyperreactivity (AHR). Our data indicate that activated ILC2s upregulate the expression of SIRPα, and the interaction between SIRPα and CD47 effectively suppresses both ILC2 proliferation and effector function. To evaluate the function of SIRPα in ILC2-mediated AHR, we combined multiple approaches including genetically modified mouse models and adoptive transfer experiments in murine models of allergen-induced AHR. Our findings suggest that the absence of SIRPα leads to the overactivation of ILC2s. Conversely, engagement of SIRPα with CD47 reduces ILC2 cytokine production and effectively regulates ILC2-dependent AHR. Furthermore, the SIRPα-CD47 axis modulates mitochondrial metabolism through the JAK/STAT and ERK/MAPK signaling pathways, thereby regulating NF-κB activity and the production of type 2 cytokines. Additionally, our studies have revealed that SIRPα is inducible and expressed on human ILC2s, and administration of human CD47-Fc effectively suppresses the effector function and cytokine production. Moreover, administering human CD47-Fc to humanized ILC2 mice effectively alleviates AHR and lung inflammation. These findings highlight the promising therapeutic potential of targeting the SIRPα-CD47 axis in the treatment of ILC2-dependent allergic asthma.

## Introduction

Allergic asthma, characterized by airway inflammation, bronchoconstriction, and airway hyperreactivity (AHR) is a global health concern with escalating prevalence attributed to recent environmental exposures [1, 2]. Despite significant therapeutic advancements, the increase in severe and difficult-to-control asthma cases necessitates urgent development of effective therapies [3, 4]. The discovery of group 2 innate lymphoid cells (ILC2s) as innate counterparts to T helper 2 (TH2) cells has garnered significant attention in understanding asthma pathogenesis and treatment. ILC2s rapidly respond to epithelial-secreted alarmins like interleukin (IL)-33, IL-25, and thymic stromal lymphopoietin (TSLP), and produce substantial amounts of type 2 cytokines (including IL-5 and IL-13), contributing to eosinophil recruitment, mucus secretion, and airway constriction [5–7]. Our research group, among others, is actively exploring ILC2s to decipher their roles in asthma pathogenesis and potential therapeutic interventions [8, 9].

The delicate control of effector functions in ILC2s and other immune cells relies on the balance between activation and inhibitory receptors [10–12]. While ILC2s share intracellular transduction receptors such as immunoreceptor tyrosine-based activation (ITAM) / inhibitory (ITIM) motifs with immune cells, their aberrant signaling in asthma exacerbations prompts investigations into therapeutic approaches blocking ITAM or stimulating ITIM [8, 13–15]. Signal regulatory protein alpha (SIRPα) is a transmembrane receptor from the ITIMs family with CD47 as its only known ligand. Recent studies have highlighted the regulatory crosstalk between myeloid and target cells through the CD47-SIRPα axis during various homeostatic processes [16]. Signaling through the CD47-SIRPα axis has been shown to regulate the maintenance of red blood cells, platelets, hematopoietic stem cells (HSCs), and neuronal development [17]. Recently, it was shown that CD47 expression on cancer cells inhibits cancer cell exclusion by myeloid cells and regulates the cytotoxic function of NK cells [18–20]. Notably, recent studies have reported the expression of SIRPα on dendritic cells, macrophages, T cells and NK cells, where it acts as an ITIM-containing receptor regulating cellular activity [21–24].

This study demonstrates the crucial role of SIRPα in regulating ILC2 function and airway inflammation. The results indicate that lung ILC2s upregulate SIRPα upon IL-33 stimulation ex vivo and in vivo. Our experiments using genetically modified mice showed that the absence of either SIRPα or CD47 leads to increased ILC2 function, development AHR and lung inflammation in both acute and chronic models of asthma. Conversely, SIRPα engagement using CD47Fc as an agonist effectively suppresses ILC2 proliferation and type 2 cytokine secretion. Mechanistically, SIRPα engagement regulates STAT3, p38, and KLF2, which affects NF-κB activity and influences ILC2 function, as treatment with CD47Fc resulted in a decrease in ILC2 bioenergetic function and mitochondrial energy production. Finally, our translational studies on human ILC2 showed that activation of SIRPα effectively inhibits ILC2 effector function by regulating mitochondrial energy. Overall, our results indicate that targeting SIRPα could be a promising therapeutic strategy for allergic asthma. This strategy could in particular involve suppressing pulmonary ILC2s by regulating their metabolism and function. Additionally, our findings suggest that SIRPα agonist activity plays a crucial role in other inflammatory diseases caused by ILC2s.

## Results

### Activated ILC2s express SIRPα

**Fig. 1:**
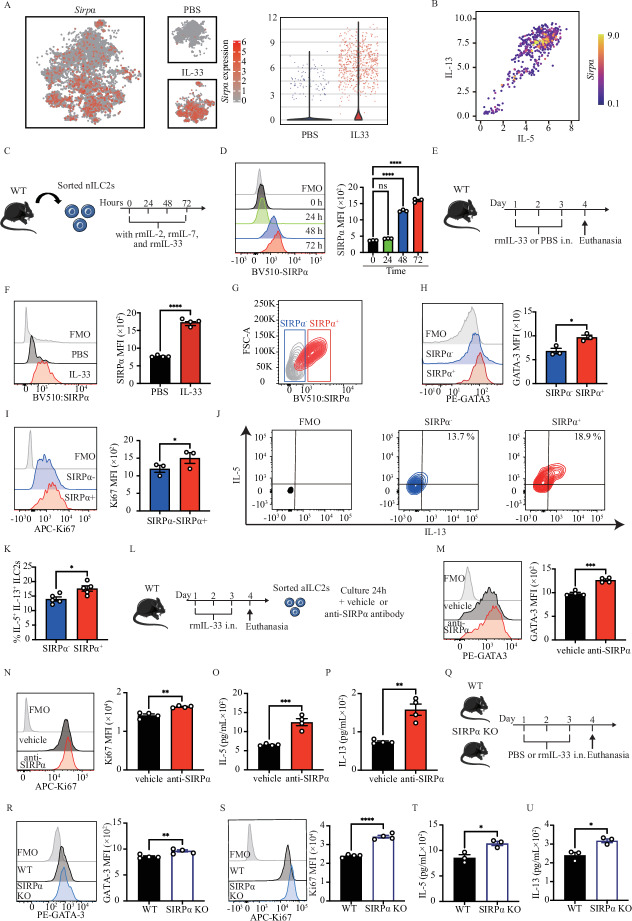
SIRPα expression is upregulated in activated ILC2s and controls ILC2s function. **A** UMAP projections of total ILC2s (left panel) and naïve ILC2s (nILC2s) versus IL-33-activated ILC2s (middle panel). Pure populations of either naïve or IL-33-activated pulmonary ILC2s were profiled by scRNAseq. Expression level of *SIRPα* is demonstrated on a gradient from gray (lowest expression) to solid red (highest expression). Violin plots comparing *Sirpα* expression level in naïve ILC2s (blue plot) versus IL-33-activated ILC2s (red plot). Dots represent cells. **B** The volcano plot shows the expression level of *Sirpα* in *Sirpα*-positive ILC2 cells based on Il5 and Il13 transcript levels. The dots represent cells, and *Sirpα* expression level is indicated by the color scale ranging from purple (lowest expression) to yellow (highest expression). **C** Pulmonary nILC2s from C57BL/6 WT mice were sorted using FACS and cultured (50 × 10^4^/ml) ex vivo in the presence of rmIL-2 (10 ng/ml), rmIL-7 (10 ng/ml), and rmIL-33 (50 ng/ml) for the indicated durations at 37 °C. Flow cytometry analysis assessed SIRPα expression over time. **D** Representative plots depict SIRPα protein expression levels at 0-, 24-, 48-, and 72 h post-culture. The gray plot represents Fluorescence-minus-one (FMO) control. Corresponding quantitation is presented as Mean Fluorescence Intensity (MFI); *n* = 3. **E** WT mice were intranasally challenged with rmIL-33 or PBS over 3 consecutive days. Lung ILC2s were isolated on day 4 and SIRPα expression in ILC2s was analyzed. **F** Representative plots of SIRPα protein expression levels in PBS and IL-33 group. The gray plot is FMO. Quantification is shown as MFI; *n* = 4. **G** Activated ILC2s were FACS-sorted based on SIRPα expression into SIRPα^-^ (bule rectangle) and SIRPα^+^ (red rectangle) populations. **H**, **I** Intranuclear protein expression levels of GATA-3 (**H**) and Ki67 (**I**) in SIRPα^-^ versus SIRPα^+^ ILC2s. Corresponding quantitation is presented as MFI; *n* = 3. **J**, **K** Frequency (%) of IL-5^+^ and IL-13^+^ ILC2s in both SIRPα^-^ and SIRPα^+^ populations; *n* = 5. **L**–**Q** WT mice received intranasal doses of rmIL-33 over 3 consecutive days. Activated ILC2s were sorted and cultured with rmIL-2 (10 ng/ml) and rmIL-7 (10 ng/ml) in the presence of either vehicle or anti-SIRPα antibody (20 μg/mL) for 24 h. **M**, **N** GATA-3 (**M**) and Ki67 (**N**) expression levels in activated ILC2s are shown. Corresponding quantitation is presented as MFI; *n* = 4. **O**, **Q** Levels of IL-5 (**O**) and IL-13 (**Q**) production in the culture supernatant were measured by LEGENDPLEX and are shown in bar graphs; *n* = 4. **R**–**V** Activated ILC2s from WT and SIRPα KO mice were cultured with rmIL-2 and rmIL-7 for 24 h. **S**, **T** Expression of GATA-3 (**S**) and Ki67 (**T**) in activated ILC2s is depicted. Corresponding quantitation is presented as MFI; *n* = 4. **U**, **V** Levels of IL-5 (**U**) and IL-13 (**V**) production in the culture supernatant were measured; *n* = 3. Data are presented as mean ± standard error of the mean (SEM) and are representative of at least 2 independent experiments. Two-tailed student’s t-test or one-way ANOVA followed by Tukey post-hoc tests were employed for statistical analysis; *< 0.05, **< 0.01, and ***< 0.001. Schematic images are sourced by an open-access license from Servier Medical Art

To evaluate the expression of SIRPα in steady-state and activated pulmonary ILC2, cohorts of WT mice were intranasally challenged with PBS or IL-33 (0.5 μg) for 3 days, with lung samples collected on day 4 for evaluation of SIRPα expression. Lung ILC2s were FACS sorted as CD45^+^, lineage^-^, CD127^+^, and ST2^+^ cells (Fig. S1A). Single-cell RNA sequencing (scRNAseq) was employed to investigate the transcriptome-level expression of *SIRPα*. The scRNAseq, visualized through UMAP plots, delineated distinct transcriptome profiles of activated and naïve ILC2s (Fig. 1A). It was found that while some cells express *Sirpα* under PBS conditions, activation with IL-33 leads to the emergence of a unique population of ILC2s that express *Sirpα* (Fig. 1A, middle panel). Furthermore, the expression level of *SIRPα* genes was measured in both naïve and IL-33-activated ILC2s (Fig. 1A, right panel). To investigate the correlation between *Sirpα* expression and *Il5* and *Il13*, we examined the expression of these cytokines in ILC2s. It is important to note the pivotal role of *Il5* and *Il13* in exacerbating AHR. Our findings showed that elevated *Il5* and *Il13* expression in ILC2s was correlated with upregulated *Sirpα* (Fig. 1B). The kinetics of SIRPα protein expression was further assessed in wild-type mice. Pure populations of naive lung ILC2s were FACS sorted and cultured ex vivo for 24, 48, and 72 h in the presence of IL-33 (Fig. 1C). Expression of SIRPα gradually increased over time with IL-33 activation, reaching peak levels 72 h post-activation (Fig. 1D). To validate these results in vivo, we assessed the expression of SIRPα protein on ILC2s in a mouse model of IL33-mediated airway inflammation. Cohorts of wild-type mice were intranasally treated with IL-33 or PBS for three consecutive days (Fig. 1E). Flow cytometry analysis on the fourth day revealed a significant upregulation of SIRPα on ILC2s in response to IL-33 (Fig. 1F).

We analyzed the expression of *Sirpα* and *Cd47* in Th1, Th2, Th17, ILC1, ILC2, and ILC3 cells using publicly available scRNAseq data generated from a mouse asthma model [25]. Our findings revealed that *Sirpα* is most highly expressed in ILC2s, while *Cd47* is expressed on a variety of immune cells (Fig. S1B). Additionally, we examined the expression of *Sirpα* and *Cd47* in immune cells, including ILC2s, within the intestinal tract and abdominal cavity of a mouse model of inflammatory bowel disease using public databases [26], and the results were comparable to the mouse asthma model (Fig. S1C). We further validated these findings using our IL-33 stimulation model, which demonstrated that ILC2s exhibited the highest SIPRα expression at the protein level, while CD47 was expressed on many different immune cells (Fig. S1D and S1E). Overall, these results suggest that targeting SIRPα on ILC2s may be a feasible approach for treating asthma.

ILC2s were further classified into SIRPα^+^ and SIRPα^-^ based on the expression of SIRPα protein (Fig. 1G). GATA-3, an activation marker for ILC2s, exhibited significantly higher expression in SIRPα^+^ ILC2s compared to their SIRPα^-^ counterparts (Fig. 1H). Furthermore, the proliferation marker Ki67 was markedly upregulated in SIRPα^+^ ILC2s (Fig. 1I). Functionally, SIRPα^+^ ILC2s exhibited a notable higher frequency of IL-5^+^/IL-13^+^ cells compared to SIRPα- counterparts (Fig. 1J, K). These results suggest that SIRPα is upregulated in activated lung ILC2 at the transcriptome and protein levels.

### SIRPα controls ILC2 activation, proliferation, and cytokine production

Having established the inducibility of SIRPα in activated ILC2s, we proceeded to investigate its impact on ILC2s functionality. WT mice were treated with IL-33 for three consecutive days (i.n.). On the fourth day, lung ILC2s were isolated and cultured in the presence of recombinant mouse (rm)IL-2, rmIL-7, and either vehicle or anti-SIRPα blocking antibody for 24 h (Fig. 1L). The absence of cytotoxic effects from anti-SIRPα antibody, we confirmed using annexin-V/DAPI staining (Fig. S1F). Evaluation of GATA-3 expression, an activation marker for ILC2s, revealed that ILC2s treated with the anti-SIRPα antibody exhibited significantly higher GATA-3 expression compared to the vehicle group (Fig. 1M). Moreover, the expression of Ki67, a marker of proliferative activity, showed a significant increase in ILC2s treated with anti-SIRPα antibody (Fig. 1N). Notably, anti-SIRPα antibody treatment significantly boosted the secretion of ILC2s effector cytokines IL-5 and IL-13 in the culture supernatant (Fig. 1O, P). Building upon these observations, we sought to elucidate the role of SIRPα in ILC2 function by utilizing SIRPα knockout (SIRPαKO) mice. Intranasal administration of IL-33 into SIRPαKO, and WT mice for three consecutive days was followed by sorting pure populations of lung ILC2s and ex vivo culture of them (Fig. 1Q). Notably, in the deficiency of SIRPα, ILC2s exhibited significantly increased expression of the activation marker GATA-3 and the proliferation marker Ki67 (Fig. 1R, S). Additionally, the secretion levels of IL-5 and IL-13 in the culture supernatant were elevated compared to WT (Fig. 1T, U). Furthermore, the cytokine production capacity per cell was examined by flow cytometry with intracellular staining of IL-5 and IL-13. The percentage of IL-5 + IL-13+ was significantly increased in ILC2s of SIRPα KO compared to ILC2s of WT (Fig. S1G). The results suggest that the deficiency of SIRPα itself results in heightened activation of ILC2s, enhancing cytokine secretion and cell proliferation. Furthermore, SIRPα may exert an inhibitory role in ILC2 activation ex vivo.

### SIRPα effectively controls ILC2-induced AHR and lung inflammation

**Fig. 2:**
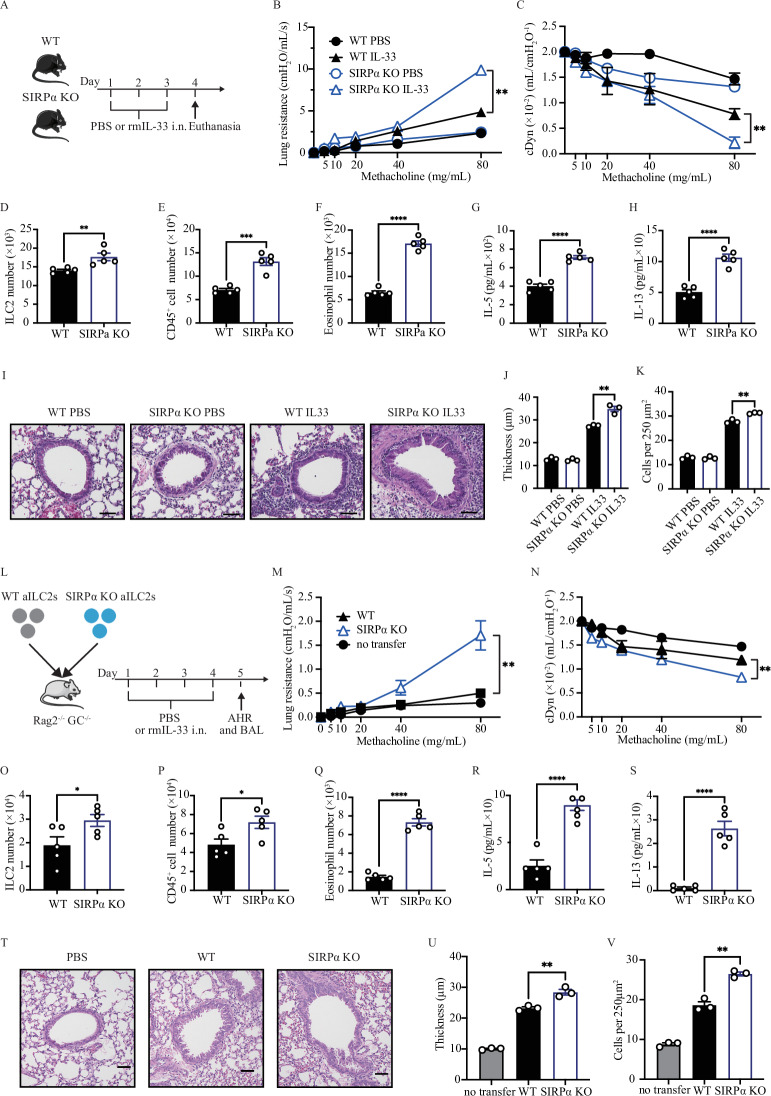
SIRPα deficiency aggravates AHR and lung inflammation. **A**–**K** Cohorts of WT and SIRPα KO mice were intranasally exposed to 0.5 µg of rmIL-33 or PBS for 3 days. On day 4, lung function, pulmonary ILC2s, BAL cellularity and cytokine levels, as well as histology were analyzed. **B, C** Lung resistance (**B**) and dynamic compliance (**C**) in response to elevating doses of methacholine are displayed; *n* = 5. **D**–**F** The total number of ILC2s per lung (**D**), total number of CD45^+^ cells (**E**) and CD45^+^, Gr-1^-^, CD11c^-^, SiglecF^+^ eosinophils (**F**) in BAL fluid are demonstrated in bar graphs; *n* = 5. **G**, **H** Levels of IL-5 (**G**) and IL-13 (**H**) in the BAL fluid were measured by LEGENDPLEX and are shown in bar graphs; *n* = 5. **I** Lung histologic sections stained with hematoxylin and eosin (H&E) are displayed; scale bars=50 µm. **J**, **K** Quantification of airway epithelium thickness **(J)** and infiltrating cells **(K)**; *n* = 3. **L**–**V** Cohorts of Rag^−/−^GC^−/−^ mice were intravenously injected with 4 × 10^5^ activated ILC2s isolated from either WT or SIRPα KO mice. Recipient mice were received 1.0 µg rmIL-33 or PBS intranasally for 4 days. On day 5, lung function, pulmonary ILC2s, BAL cellularity and cytokine levels, as well as histology were analyzed. **M**, **N** Lung resistance (**M**) and dynamic compliance (**N**) in response to elevating doses of methacholine are depicted; *n* = 5. **O**–**S** The total number of ILC2s per lung (**O**), total number of CD45^+^ cells (**P**) and CD45^+^, Gr-1^-^, CD11c^-^, SiglecF^+^ eosinophils (**Q**) in BAL fluid are demonstrated in bar graphs; *n* = 5. **R**, **S** Levels of IL-5 (**R**) and IL-13 (**S**) in the BAL fluid are shown in bar graphs; *n* = 5. **T** Lung histologic sections stained with hematoxylin and eosin (H&E) are presented; scale bars=50 µm. **U, V** Quantification of airway epithelium thickness (**U**) and infiltrating cells (**V**); *n* = 3. Data are presented as means ± SEM and are representative of at least 2 independent experiments. Two-tailed student’s t-test or one-way ANOVA followed by Tukey post-hoc tests were employed for statistical analysis; *< 0.05, **< 0.01, ***< 0.001, and ****< 0.0001. Schematic images are sourced by an open access license from Servier Medical Art

In our pursuit of elucidating the role of SIRPα signaling in the regulation of AHR, we employed SIRPα KO mice. Cohorts of WT and SIRPα KO mice underwent intranasal administration of IL-33 or PBS for three consecutive days (Fig. 2A). On day 4, we measured lung resistance and dynamic compliance, followed by the analysis of BAL samples (Fig. S2). SIRPα KO mice exhibited significantly higher pulmonary resistance compared to WT mice (Fig. 2B), with an exacerbated response observed in dynamic compliance results (Fig. 2C). The number of ILC2s in SIRPα KO mice was significantly increased compared to WT mice (Fig. 2D). SIRPα deficiency significantly intensified lung inflammation, as demonstrated by the increase in CD45^+^ cells and eosinophils (Fig. 2E, F). Furthermore, levels of IL-5 and IL-13 were significantly elevated in SIRPα KO mice compared to their WT counterparts (Fig. 2G, H). Histological analysis of lung tissue (Fig. 2I) supported these results, showing that IL-33 challenges significantly increased epithelial thickness (Fig. 2J) and inflammatory cell count (Fig. 2K) in SIRPα KO mice compared to WT mice. To address potential confounding effects that may arise from SIRPα knockout on the lung microenvironment, we evaluated ILC2-induced airway inflammation in alymphoid mice. We transferred pure populations of ILC2s that were sorted from both WT and SIRPα KO mice to Rag2^−/−^ GC^−/−^ mice (Fig. 2L). After three days of IL-33 intranasal administration, mice were given either SIRPα KO ILC2s or WT ILC2s. On the fourth day, mice receiving SIRPα KO ILC2s showed significantly higher lung resistance and exacerbated dynamic compliance compared to those receiving WT ILC2s (Fig. 2M, N). The number of ILC2s in mice receiving SIRPα KO ILC2s was significantly increased compared to those receiving WT ILC2s (Fig. 2O). Transferring SIRPα KO ILC2s significantly intensified lung inflammation, as demonstrated by the increase in CD45+ cells and eosinophils (Fig. 2P, Q). Additionally, levels of IL-5 and IL-13 were significantly elevated in the transferred SIRPα KO ILC2s mice compared to the transferred WT ILC2s mice (Fig. 2R, S). Histological analysis of lung tissue (Fig. 2T) supported the results, indicating that IL-33 challenges significantly increased epithelial thickness (Fig. 2U) and inflammatory cell count (Fig. 2V) in mice receiving SIRPα KO ILC2s compared to those receiving WT ILC2s.

These findings highlight the crucial role of SIRPα in modulating ILC2-induced AHR and lung inflammation.

### SIRPα regulates acute and chronic allergen-induced AHR

**Fig. 3:**
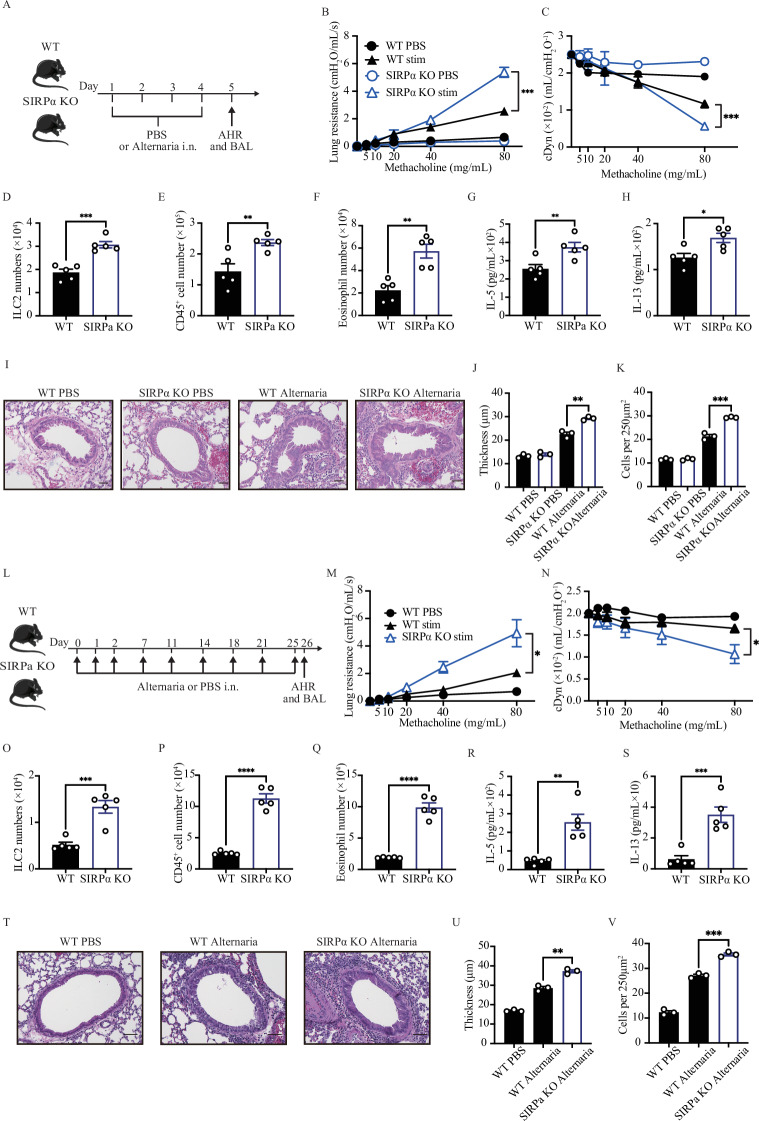
SIRPα deficiency exacerbates *A. alternata*-induced AHR in acute and chronic models. **A**–**L** WT and SIRPα KO mice were intranasally received 100 µg of *A. alternata* on days 1–4. On day 5, AHR and lung inflammation were assessed. **B**, **C** Lung resistance (**B**) and dynamic compliance (**C**) in response to elevating doses of methacholine are displayed; *n* = 5. **D**–**G** The total number of ILC2s per lung (**D**), total number of CD45^+^ cells (**E**) and eosinophils (**F**) in BAL fluid are demonstrated in bar graphs; *n* = 5. **G**, **H** Levels of IL-5 (**G**) and IL-13 (**H**) in the BAL fluid were measured by LEGENDPLEX and shown in bar graphs; *n* = 5. **I** Lung histologic sections stained with hematoxylin and eosin (H&E) are illustrated; scale bars=50 µm. **J, K** Quantification of airway epithelium thickness (**J**) and infiltrating cells (**K**); *n* = 3. **L** A cohort of WT and SIRPα KO mice were challenged intranasally with *Alternaria alternata* (*A. alternata*) or PBS for first three days. They were then challenged on day 7, 11, 14, 18, 21, and 25. **M**, **N** Lung resistance (**M**) and dynamic compliance (**N**) in response to elevating doses of methacholine; *n* = 5. **O**–**Q** Total number of ILC2s per lung (**O**), and total number of CD45^+^ cells (**P**) and eosinophils (**Q**) in BAL fluid have been demonstrated in bar graphs; *n* = 5. **R**, **S** Levels of IL-5 (**R**) and IL-13 (**S**) in the BAL fluid; *n* = 5. **T** Lung histologic sections stained with hematoxylin and eosin (H&E) are presented; scale bars=50 µm. **U**, **V** Quantification of airway epithelium thickness (**U**) and infiltrating cells (**V**); *n* = 3. Data are presented as means ± SEMs and are representative of at least 2 independent experiments. Two-tailed student’s t-test or one-way ANOVA followed by Tukey post-hoc tests; *p < 0.05, **p < 0.01, and ***p < 0.001. Schematic images are sourced by an open-access license from Servier Medical Art

To further confirm that SIRPα deficiency exacerbates AHR, we employed a clinically relevant pulmonary inflammatory disease model using *Alternaria alternata (A. alternata)*, a common fungus associated with allergic disease. WT and SIRPα KO mice were exposed to 100 µg of *A. alternata* or PBS i.n. over days 1–4. On day 5, we analyzed lung function, pulmonary ILC2 number, BAL-infiltrating cell count, and histology (Fig. 3A). Consistent with our previous findings, the lung resistance of SIRPα KO mice was significantly higher, and their dynamic compliance was considerably improved compared to WT mice (Fig. 3B, C). Additionally, SIRPα KO mice showed a substantial increase in the total number of pulmonary ILC2s (Fig. 3D). Furthermore, the numbers of CD45^+^ cells and eosinophils in BAL samples of SIRPα KO mice were significantly higher than those in WT mice (Fig. 3E, F). The levels of IL-5 and IL-13 in the BAL fluid significantly increased in SIRPα KO mice (Fig. 3G, H). Histological analysis of the lungs showed that the thickness of the epithelium and inflammatory cell recruitment considerably increased in SIRPα KO mice (Fig. 3I–K). Furthermore, we established a chronic mouse model of AHR with *A. alternata* in WT and SIRPα KO mice (Fig. 3L). The study demonstrated that deficiency in SIRPα significantly worsened AHR, increased ILC2 counts in the lungs, and raised CD45^+^ cell and eosinophil count in BAL fluid. Additionally, levels of IL-5 and IL-13 in BAL were significantly higher in SIRPα KO mice compared to WT counterparts (Fig. 3L–V). This thorough analysis suggests that SIRPα plays a pivotal role in improving both acute and chronic allergen-induced AHR.

### SIRPα modulates ILC2 mitochondrial respiration by suppressing the NF-κB pathway

**Fig. 4:**
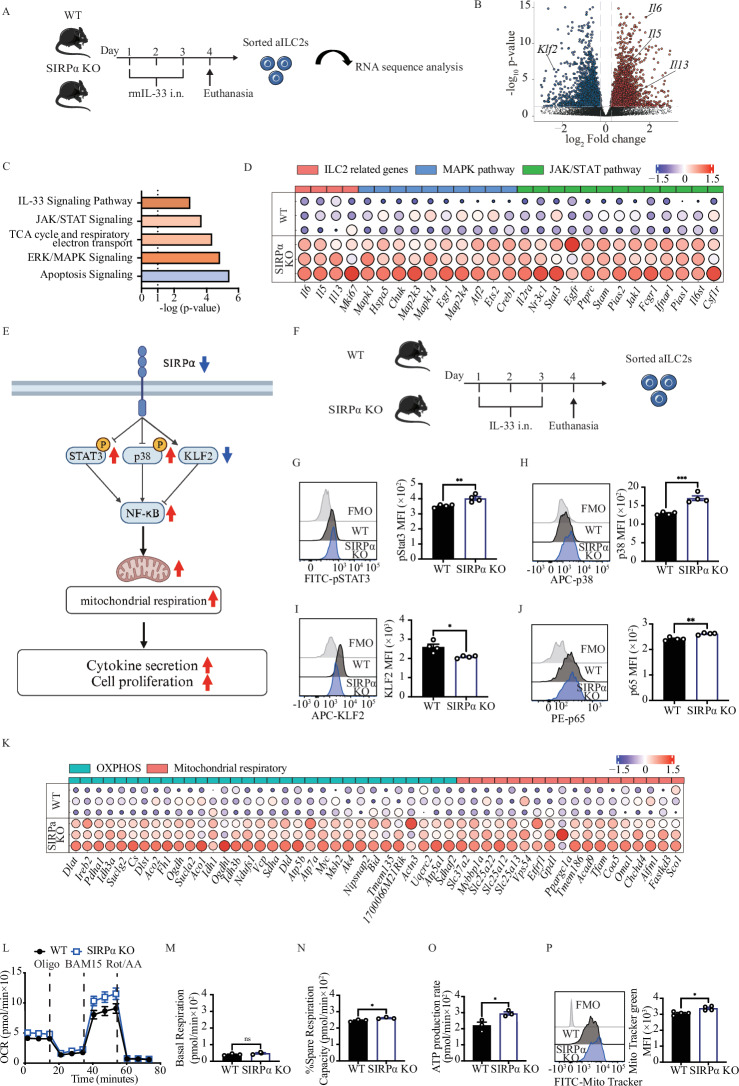
SIRPα modulates ILC2 mitochondrial respiration via NF-κB pathways. **A**–**P** Cohorts of WT and SIRPα KO mice were intranasally challenged with rmIL-33 over 3 consecutive days. On day 4, lung ILC2s were isolated and cultured with rmIL-2 and rmIL-7 for 24 h. **B** Total RNA was extracted to perform a bulk transcriptomic analysis. Volcano plots represent differentially expressed genes. **C** Gene set enrichment analysis by Ingenuity Pathway Analysis (IPA) depicting critical pathways regulated in SIRPα-deficient ILC2s. **D** Dot plot representation of selected critical genes involved in ILC2 related genes, MAPK, and JAK/STAT pathways. Dot size is indicative of the total gene expression level. **E** Overview of downstream SIRPα signaling elements. **F**–**I** Cohorts of WT and SIRPα KO mice were challenged intranasally for 3 days with rmIL-33. On day 4, lung ILC2s were isolated and cultured with rmIL-2, rmIL-7 for 24 h. Representative histogram of protein expression of pSTAT3 (**G**), p38 (**H**), KLF2 (**I**), and p65 (**J**). Corresponding quantitation is presented as MFI; *n* = 4. Corresponding quantitation is presented for each protein as MFI; *n* = 4. **K** Dot plot representation of selected critical genes involved in OXPHOS and Mitochondrial respiratory pathways. Dot size is indicative of the total gene expression level. **L**–**O** Mitochondrial respiratory profile showing oxygen consumption rates (OCR) in response to sequential injections of Oligomycin (ATP synthase inhibitor), BAM15 (mitochondrial uncoupler), and Rotenone + antimycin A (complex I and II inhibitors). Key parameters of mitochondrial function, including basal respiration (**M**), spare respiratory capacity (**N**), and ATP production rate (**O**) are presented; *n* = 3. **P** Mitochondrial sizes were assessed using Mito Tracker green and are shown in plot graphs. Data are presented as mean +/− SEM and are representative of at least 2 experiments. Two-tailed student’s t-test was employed for statistical analysis; *< 0.05, **< 0.01, ***< 0.001, and ns= non-significant. Schematic images are sourced by an open-access license from Servier Medical Art

In recent years, a growing body of evidence has implicated mitochondrial respiration in influencing the cytokine secretion capacity of ILC2s [27, 28]. To unravel the metabolic changes associated with SIRPα deficiency in ILC2s, we isolated pulmonary ILC2s from mice subjected to intranasal IL-33 challenges for 3 days. Subsequently, RNA sequencing (RNAseq) was performed on ILC2s following an 18 h culture with rmIL-2 and rmIL-7 (Fig. 4A). We observed 4696 differentially expressed genes (DEGs) induced by SIRPα deficiency. Notably, the lack of SIRPα resulted in the upregulation of genes encoding TH2 cytokines, such as *Il6*, *Il5*, and *Il13* concomitant with the downregulation of *Klf2* (Fig. 4B). To validate the association between SIRPα signaling and these pathways, Ingenuity Pathways Analysis (IPA) was employed, demonstrating upregulation of the IL-33 signaling, JAK/STAT signaling, TCA cycle and respiratory electron transfer, and ERK/MAPK signaling pathways along with the downregulation of apoptosis signaling pathway in SIRPα KO ILC2s (Fig. 4C). Furthermore, SIRPα deficiency upregulated genes related to the key molecules in the mitogen-activated protein kinase (MAPK) pathway (*Mapk14)* and Janus kinase/signal transducer and activator of transcription (JAK/STAT) pathway (*Jak1, Stat3*) (Fig. 4D). Literature reviews have identified the JAK/STAT and ERK/MAPK pathways as regulators of NF-κB [24, 29–32]. KLF2 has also been identified as an inhibitor of NF-κB [33, 34]. Additionally, NF-κB has been reported to regulate mitochondrial energy production [29]. Based on the results obtained in the IPA, a mechanism to control NF-κB is hypothesized, as shown in Fig. 4E. The expression levels of each protein were examined. To confirm the transcriptomics results, we examined the expression of crucial proteins involved in aforementioned signaling pathways within activated lung ILC2s from WT and SIRPα KO mice (Fig. 4F). Our findings indicate that SIRPα deficiency in ILC2s resulted in a significant increase in phosphorylated STAT3 (pSTAT3), p38, and p65, as well as a decrease in KLF2 (Fig. 4G–J). At the metabolic level, our transcriptomic analysis revealed that loss of SIRPα in ILC2s induced an overall upregulation of OXPHOS and mitochondrial respiratory gene signatures (Fig. 4K). In particular, TCA cycle enzymes citrate synthase (*Cs*), aconitase (*Aco1, Aco2*), isocitrate dehydrogenase (*Idh1, Idh3a, Idh3b*), oxoglutarate dehydrogenase (*Ogdh*), succinate-CoA ligase GDP-forming subunit beta (*Suclg2*), succinate dehydrogenase (*Sdha*) and fumarate hydratase (*Fh1*) were all increased in ILC2s lacking SIRPα. To confirm our transcriptomic results, we measured the impact of SIRPα on mitochondrial respiration in ILC2s by measuring live oxidative metabolism (Fig. 4L). Although basal respiration remained consistent between WT and SIRPα KO ILC2s, loss of SIRPα significantly elevated spare respiratory capacity and ATP production rates (Fig. 4M–O). Additionally, we observed higher mitochondrial activation in SIRPα KO ILC2s compared to the control group, as measured by Mito tracker green expression (Fig. 4P). To investigate the causal relationship between the SIRPα-CD47 axis and mitochondrial function in the regulation of ILC2 activity, we administered the mitochondrial complex I inhibitor BAY 87-2243 to ILC2s isolated from SIRPα KO and examined the impact on cytokine production capacity and ILC2 activation [35, 36]. The absence of cytotoxic effects of BAY 87-2243 was confirmed using annexin-V/DAPI staining (Fig. S3A). The results demonstrated that ILC2s treated with BAY87-2243 exhibit a markedly diminished expression of GATA-3, a marker of ILC2 activation, and Ki67, a marker of cellular proliferation, in comparison to the vehicle group (Fig. S3B and S3C). Additionally, the secretion levels of IL-5 and IL-13 in the culture supernatant were decreased compared to the vehicle group (Fig. S3D and S3E). Collectively, these findings suggest that SIRPα exerts control over cytokine production and cell proliferative capacity in ILC2 cells by regulating mitochondrial oxidative energy production.

### CD47 is upregulated in activated ILC2s and plays a crucial role in limiting cellular effector function

**Fig. 5:**
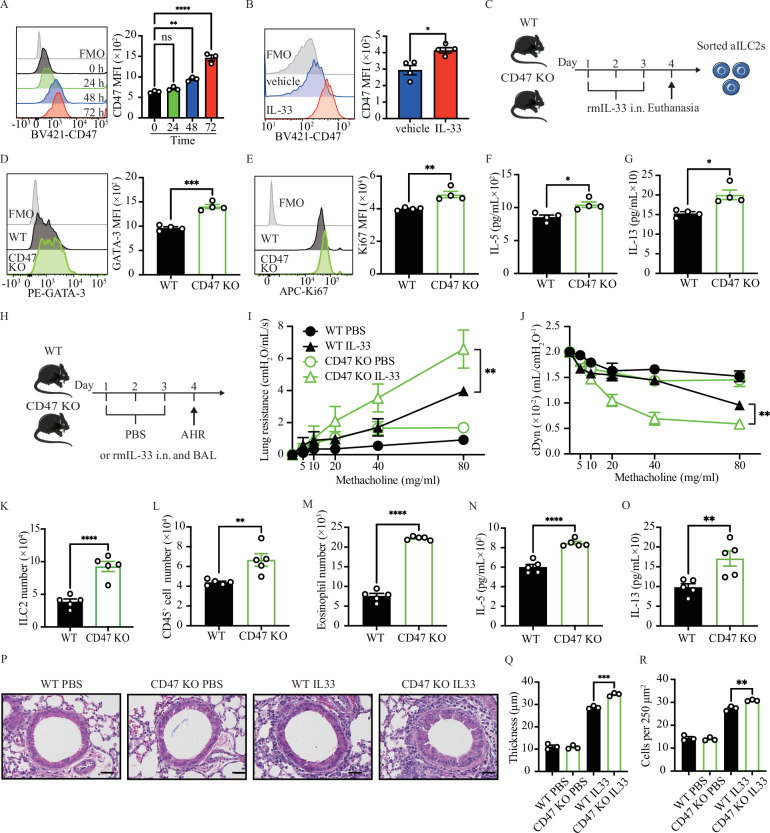
Lack of CD47 increases ILC2 function and aggravates AHR. **A** Pulmonary nILC2s from C57BL/6 WT mice were sorted using FACS and cultured (50 × 10^4^/ml) ex vivo in the presence of rmIL-2 (10 ng/ml), rmIL-7 (10 ng/ml), and rmIL-33 (50 ng/ml) for the indicated durations at 37 °C. Flow cytometry analysis assessed CD47 expression over time. Representative plots depict CD47 protein expression levels at 0-, 24-, 48-, and 72 h post-culture. The gray plot represents Fluorescence-minus-one (FMO) control. Corresponding quantitation is presented as Mean Fluorescence Intensity (MFI); *n* = 3. **B** WT mice were intranasally challenged with rmIL-33 or PBS over 3 consecutive days. Lung ILC2s were isolated on day 4 and CD47 expression in ILC2s was analyzed. Representative plots of CD47 protein expression levels in PBS and IL-33 group. The gray plot is FMO. Quantification is shown as MFI; *n* = 4. **C**–**G** Activated ILC2s were sorted from WT and CD47 KO mice following three intranasal challenges with rm-IL-33 (0.5 μg/mouse). Sorted cells were incubated with rmIL-2 and rmIL-7 for 24 h. **D, E** Expression levels of GATA-3 (**D**) and Ki67 (**E**) in activated ILC2s are presented. Corresponding quantitation is presented as MFI; *n* = 4. **F**, **G** Levels of IL-5 (**F**) and IL-13 (**G**) production in the culture supernatant were measured; *n* = 4. **H**–**R** A cohort of WT and CD47 KO mice received were intranasally exposed to 0.5 µg of rmIL-33 or PBS for 3 days. On day 4, lung function, pulmonary ILC2s, BAL cellularity, and cytokine levels, as well as histology were analyzed. **I, J** Lung resistance (**I**) and dynamic compliance (**J**) in response to elevating doses of methacholine; *n* = 5. **K**–**M** Total number of ILC2s per lung (**K**) as well as total number of CD45^+^ cells (**L**) and eosinophils (**M**) in BAL fluid have been demonstrated in bar graphs; *n* = 5. **N**, **O** Levels of IL-5 (**N**) and IL-13 (**O**) in the BAL fluid were measured by Legendplex and shown in bar graphs; *n* = 5. **P** Lung histologic sections stained with hematoxylin and eosin (H&E); scale bars=50 µm. **Q**, **R** Quantification of airway epithelium thickness (**Q**) and infiltrating cells (**R**); *n* = 3. Data are presented as means ± SEM and are representative of at least 2 independent experiments. Statistical analysis was performed using two-tailed Student’s t-test; *< 0.05, **< 0.01, ***< 0.001, and ****< 0.0001. Schematic images are sourced by an open access license from Servier Medical Art

To further explore the role of CD47-SIRPα axis in ILC2s, we examined its expression in naïve and activated ILC2s. Pure populations of naive lung ILC2s were FACS sorted and cultured ex vivo for 24, 48, and 72 h in the presence of IL-33. Expression of CD47 gradually increased over time with IL-33 activation, reaching peak levels 72 h post-activation (Fig. 5A). To validate this result in vivo, we assessed the expression of CD47 proteins on ILC2s in a mouse model of IL33-mediated airway inflammation. Cohorts of wild-type mice were intranasally treated with IL-33 or PBS for three consecutive days. Flow cytometry analysis on the fourth day revealed a significant upregulation of CD47 on ILC2s in response to IL-33 (Fig. 5B). To investigate the role of CD47 in ILC2s, we utilized WT mice and CD47 knockout (CD47 KO) mice. Intranasal administrations of IL-33 into WT and CD47 KO mice for three consecutive days were followed by sorting and ex vivo culture of pure populations of lung ILC2s (Fig. 5C). Notably, in the absence of CD47, ILC2s exhibited significantly increased expression of the activation marker GATA-3 and the proliferation marker Ki67 (Fig. 5D, E). Additionally, the secretion levels of IL-5 and IL-13 in the culture supernatant were elevated in CD47 KO mice compared to WT counterparts (Fig. 5F, G). Furthermore, the cytokine production capacity per cell was examined by flow cytometry with intracellular staining of IL-5 and IL-13. The percentage of IL-5^+^IL-13^+^ was significantly increased in ILC2s of CD47 KO compared to ILC2s of WT (Fig. S3F). These results suggest that the loss of the CD47 results in enhanced activation of ILC2s, elevating cytokine secretion and cell proliferation. To elucidate the impact of CD47 deficiency on AHR and lung inflammation, we examined AHR indices in CD47 KO mice. Both WT and CD47 KO mice underwent intranasal administration of IL-33 (0.5 μg) or PBS for 3 consecutive days (Fig. 5H). On day 4, we directly measured lung resistance and dynamic compliance, as well as flow cytometry analyses on BAL and lung samples. Similar to SIRPα KO mice, the pulmonary resistance was significantly lower in WT mice compared to CD47 KO mice (Fig. 5I, J). The number of ILC2s in CD47 KO mice significantly increased compared to WT mice (Fig. 5K). The lack of CD47 expression resulted in a significant exacerbation of lung inflammation, as indicated by increased CD45^+^ cells and eosinophils (Fig. 5L, M). Additionally, elevated levels of IL-5 and IL-13 were observed in CD47 KO mice compared to WT mice (Fig. 5N, O). Histological analysis of the lungs (Fig. 5P) corroborated these findings, demonstrating that IL-33-induced thickening of the epithelium (Fig. 5Q) and increase in inflammatory cell count (Fig. 5R) were more pronounced in CD47 KO mice compared to WT mice. These results suggest that CD47, the sole ligand for SIRPα, is expressed on ILC2s and is crucial for limiting their function.

### Endogenous and exogenous CD47 regulate ILC2 function via activation of SIRPα pathway

**Fig. 6:**
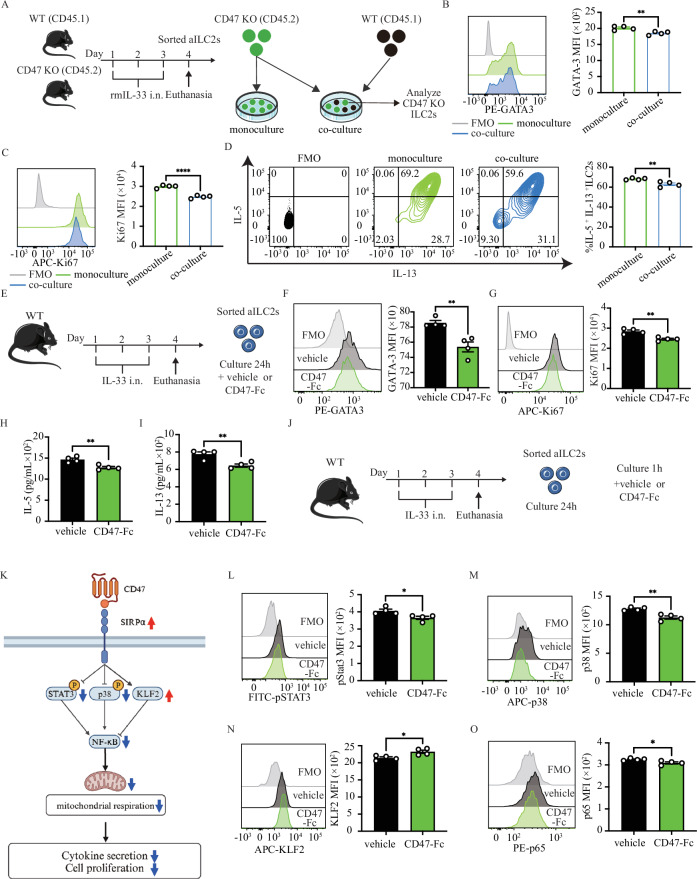
CD47 administration controls ILC2 function via activating SIRPα signaling. **A**)WT (CD45.1) and CD47 KO (CD45.2) mice were intranasally exposed to 0.5 μg rmIL-33 for three days, and lung ILC2s were isolated on day four. Two groups were formed: one consisting ILC2s from only CD47 KO mice (monoculture group), and another consisting of ILC2s from both CD47 KO (CD45.2) and WT (CD45.1) mice, which were co-cultured in a 1:1 ratio (co-culture group) and incubated for 24 h. The function of CD47 KO-derived ILC2 in each group was then evaluated by FACS. **B**, **C** Expression levels of GATA-3 (**B**) and Ki67 (**C**) in activated ILC2s are presented. Corresponding quantitation is presented as MFI; *n* = 4. **D** Frequency (%) of IL-5^+^ and IL-13^+^ ILC2s in both monoculture and co-culture groups; *n* = 4. **E**–**I** WT mice received intranasal doses of rmIL-33 over 3 consecutive days. Activated ILC2s were sorted and cultured with rmIL-2 (10 ng/ml) and rmIL-7 (10 ng/ml) in the presence of either vehicle or CD47-Fc (20 μg/mL) for 24 h. **F**, **G** GATA-3 (**F**) and Ki67 (**G**) expression levels in activated ILC2s are shown. Corresponding quantitation is presented as MFI; *n* = 4. **H**, **I** Levels of IL-5 (**H**) and IL-13 (**I**) production in the culture supernatant were measured by LEGENDPLEX and are shown in bar graphs; *n* = 4. **J** WT mice were treated with rmIL-33 by intranasal injection for three days in a row. Activated ILC2 cells were sorted and cultured with rmIL-2 (10 ng/ml) and rmIL-7 (10 ng/ml). The following day, vehicle or CD47-Fc (20 μg/mL) was added to the culture wells and analyzed by FACS after one hour. **K** Overview of downstream SIRPα signaling elements. **L**–**O** Representative histogram of protein expression of pSTAT3 (**L**), p38 (**M**), KLF2 **(N**), and p65 (**O**). Corresponding quantitation is presented for each protein as MFI; *n* = 4. Data are presented as means ± SEM and are representative of at least 2 independent experiments. Two-tailed student’s t-test or one-way ANOVA followed by Tukey post-hoc tests were employed for statistical analysis; *< 0.05, **< 0.01, ***< 0.001, and ****< 0.0001. Schematic images are sourced by an open access license from Servier Medical Art

To investigate a therapeutic approach of targeting SIRPα-CD47 pathway, we questioned whether endogenous CD47 on ILC2s can downregulate ILC2 function by binding to SIRPα on ILC2. CD47 KO (CD45.2) and WT (CD45.1) mice were intranasally treated with IL-33 for three consecutive days, and pulmonary ILC2s were isolated on the fourth day. Two groups were formed: one consisting solely of ILC2s from only CD47 KO mice (monoculture), and another group consisting of ILC2 from both CD47 KO (CD45.2) and WT (CD45.1) mice, which were co-cultured in a 1:1 ratio (co-culture group) and incubated for 24 h. The function of CD47 KO-derived ILC2 in each group was evaluated by FACS (Fig. 6A and S3G). The expression of the ILC2 activation marker GATA-3 and the proliferation marker Ki67 significantly decreased in the group of co-culturing WT and CD47 KO ILC2s compared to CD47 KO monoculture ILC2s (Fig. 6B, C). Additionally, the percentage of ILC2s positive for both IL-5 and IL-13 was reduced in the co-culture group compared to the monoculture group (Fig. 6D).

To explore the potential of exogenous CD47 to further limit the ILC2 function, we designed a set of experiments using CD47-Fc protein. WT mice were treated with IL-33 (i.n.) for three consecutive days. On the fourth day, lung ILC2s were isolated and cultured in the presence of rmIL-2, rmIL-7, and either vehicle or CD47-Fc for 24 h (Fig. 6E). The absence of cytotoxic effects of CD47-Fc was confirmed using annexin-V/DAPI staining (Fig. S3H). Evaluation of GATA-3 expression revealed that ILC2s treated with CD47-Fc displayed significantly lower GATA-3 expression compared to the vehicle group (Fig. 6F). Moreover, the expression of Ki67 showed a decrease in ILC2s treated with CD47-Fc (Fig. 6G). Notably, CD47-Fc treatment significantly inhibited IL-5 and IL-13 secretion (Fig. 6H, I). These results indicated that although endogenous CD47 on ILC2s is able to limit their function, exogenous CD47 is required to further mitigate the ILC2 proliferation and effector function.

Next, we explored the signaling of SIRPα following ligation to CD47. WT lung aILC2s were incubated with CD47-Fc for 1 h and the downstream signaling proteins were measured (Fig. 6J). It was hypothesized that CD47 therapy would activate SIRPα signaling, as demonstrated in Fig. 6K. Contrary to SIRPα KO mice, ILC2s treated with the CD47-Fc showed significant decreases in pSTAT3, p38, and p65, along with an increase in KLF2 (Fig. 6L–O). These findings show that signaling of SIRPα-CD47 axis regulates ILC2 function and proliferation via downregulation of JAK-STAT, MAPK, and NF-κB pathways.

### CD47-Fc treatment attenuates allergen induced AHR

**Fig. 7:**
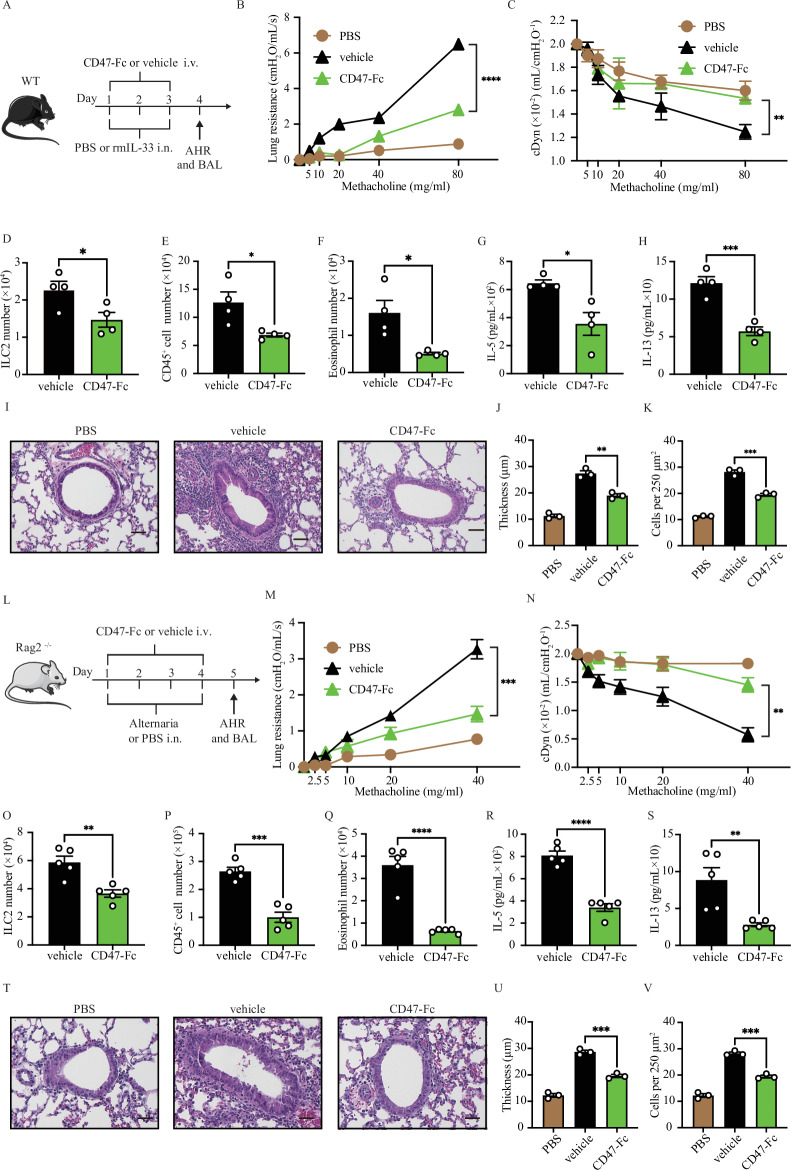
CD47 administration alleviates IL-33- and *A. alternata*-induced AHR. **A**–**K** WT mice received intravenous injections of 50 μg CD47-Fc or vehicle and were intranasally exposed to 0.5 µg of rmIL-33 or PBS for 3 days. On day 4, lung function, pulmonary ILC2s, BAL cellularity and cytokine levels, as well as histology were analyzed. **B, C** Lung resistance (**B**) and dynamic compliance (**C**) in response to increasing doses of methacholine are depicted; *n* = 4. **D**–**F** Total number of ILC2s per lung (**D**), total number of CD45^+^ cells (**E**) and eosinophils (**F**) in BAL fluid are demonstrated in bar graphs; *n* = 4. **G**, **H** Levels of IL-5 (**G**) and IL-13 (**H**) in the BAL fluid were measured by LEGENDPLEX and are shown in bar graphs; *n* = 4. **I** Lung histologic sections stained with hematoxylin and eosin (H&E) are displayed; scale bars=50 µm. **J**–**K** Quantification of airway epithelium thickness (**J**) and infiltrating cells (**K**); *n* = 3. **L**–**V** Rag2^−/−^ mice received intravenous injections of 50 μg CD47-Fc or vehicle and was intranasally exposed to 100 µg of *A. alternata* on days 1–4. On day 5, AHR and lung inflammation were assessed. **M, N** Lung resistance (**M**) and dynamic compliance (**N**) in response to elevating doses of methacholine; *n* = 5. **O**–**Q** Total number of ILC2s in the lung (**O**), and the total number of CD45^+^ cells (**P**), eosinophils (**Q**) in BAL fluid; *n* = 5. **R**, **S** Levels of IL-5 (**R**) and IL-13 (**S**) in the BAL fluid; *n* = 5. **T** Lung histology; scale bars=50 µm. **U**, **V** Quantification of airway epithelium thickness and infiltrating cells; *n* = 3. Data are presented as means ± SEMs and are representative of at least 2 independent experiments. Two-tailed student’s t-test or one-way ANOVA followed by Tukey post-hoc tests; *p < 0.05, **p < 0.01, and ***p < 0.001. Schematic images are sourced by an open-access license from Servier Medical Art

We sought to determine the efficacy of SIRPα activation in mitigating IL-33-induced AHR. Cohorts of WT mice were intranasally received IL-33 (0.5 μg) or PBS and treated with either vehicle (control) or CD47-Fc (50 μg) intravenously for 3 consecutive days (Fig. 7A). On the fourth day, we directly measured lung resistance and dynamic compliance in anesthetized tracheostomized mice to assess lung function. Following AHR assessment, we cannulated the trachea and collected bronchoalveolar lavage fluid (BAL) for subsequent flow cytometry analysis. The pulmonary resistance was significantly higher in the control mice compared to the CD47-Fc-treated mice (Fig. 7B). The CD47-Fc-treated group showed an improved dynamic compliance compared to the controls (Fig. 7C). Flow cytometry analysis revealed a notable reduction in the number of ILC2s in CD47-Fc-treated mice compared to controls (Fig. 7D). CD47-Fc treatment significantly alleviated lung inflammation, evidenced by decreased CD45^+^ cells and eosinophils (Fig. 7E, F, Fig. S1C). Additionally, levels of ILC2 cytokines IL-5 and IL-13 were significantly reduced in CD47-Fc-treated mice compared to controls (Fig. 7G, H). Histological examination of the lungs (Fig. 7I) supported these results, showing that IL-33 challenge significantly thickened the epithelium (Fig. 7J) and increased the number of inflammatory cells (Fig. 7K) in the control group compared to the CD47-Fc-treated cohort. To explore the impact of CD47-Fc in ILC2-mediated AHR in response to a clinically relevant allergen, we utilized *A. alternata*-induced AHR in Rag2^−/−^ mice. WT mice were administered i.v. injections of CD47-Fc (50 µg) or vehicle and exposed to 100 µg of *A. alternata* or PBS i.n. over days 1–4. On day 5, we analyzed lung function, pulmonary ILC2 number, BAL-infiltrating cell count, and histology (Fig. 7L). Rag2^−/−^ mice treated with CD47-Fc showed a significant reduction in lung resistance and improved dynamic compliance compared to controls (Fig. 7M, N). This effect was supported by the decreased numbers of pulmonary ILC2, CD45^+^ cells, and eosinophils in BAL fluid, as well as lower levels of IL-5 and IL-13 in the BAL fluid of the CD47-Fc-treated group (Fig. 7O–S). Histological analysis of the lungs showed a significant decrease in epithelium thickness and the number of inflammatory cells in the CD47-Fc treatment group (Fig. 7T–V). These observations suggest that Exogenous CD47 can prevent the development of ILC2-mediated AHR.

### SIRPα-CD47 axis regulates human ILC2s and reduces AHR and lung inflammation in humanized mice

**Fig. 8:**
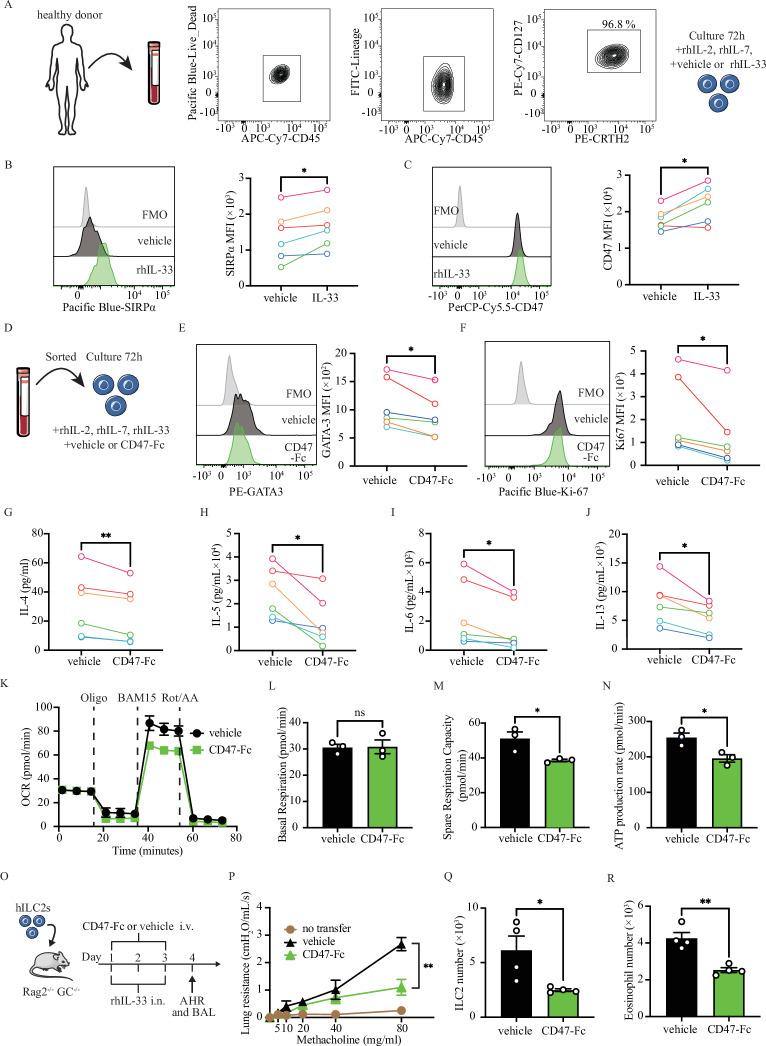
SIRPα regulates human ILC2 function and proliferation. **A**–**C** Human ILC2s (CD45^+^, Lineage^-^, CRTH2^+^, CD127^+^) were freshly isolated from peripheral blood mononuclear cells (PBMCs) of healthy donors and cultured with recombinant human (rh)IL-2 and rhIL-7 (both 20 ng/mL) with or without rhIL-33 (100 ng/mL) for 72 h. **B** Representative histogram plots of SIRPα expression in human ILC2s (hILC2s) and corresponding quantitation presented as MFI; *n* = 6. **C** Representative histogram plots of CD47 expression in human ILC2s (hILC2s) and corresponding quantitation presented as MFI; *n* = 6. **D** Freshly isolated hILC2s from healthy donors were cultured in the presence of rhIL-2, rhIL-7 (both 20 ng/mL), and rhIL-33 (100 ng/mL), with vehicle or CD47-Fc (20 µg/mL) for 72 h. **E**, **F** Representative histogram plots of intranuclear GATA-3 (**E**) and Ki67 (**F**) expression levels and corresponding quantitation presented as MFI; *n* = 6. **G**–**J** Levels of IL-4 (**G**), IL-5 (**H**), IL-6 (**I**), and IL-13 (**J**) in the culture supernatants following treatment with vehicle or CD47-Fc (20 µg/mL); *n* = 6. **K** Mitochondrial respiratory profile showing oxygen consumption rates (OCR) in response to sequential injections of Oligomycin (ATP synthase inhibitor), BAM15 (mitochondrial uncoupler), and Rotenone + antimycin A (complex I and II inhibitors). Corresponding key parameters of mitochondrial function, including basal respiration (**L**), spare respiratory capacity (**M**), and ATP production rate (**N**); *n* = 3. **O** Schematic representation of the AHR induction in alymphoid mice adoptively transferred with human ILC2s. Freshly CD45^+^ Lineage^-^ CRTH2^+^ CD127^+^ hILC2s were isolated from PBMCs of healthy subjects and cultured (5 × 10^5^/mL) in rhIL-2, rhIL-7, (both 20 ng/mL) with decreasing doses of rhIL-33 (from 100 ng/mL to 10 ng/mL) for 10 days. 2 × 10^5^ hILC2s were then transferred intravenously to Rag^−/−^GC^−/−^ recipient mice, followed by intravenous injections of 25 μg human CD47-Fc or vehicle and intranasal exposure to 1 µg of rhIL-33 or PBS for 3 days. On day 4, lung function, lung ILC2s and BAL cellularity were analyzed. **P** Lung resistance in response to increasing doses of methacholine; *n* = 4. **Q** Total number of hILC2s per lung, *n* = 4. **R** Total number of BAL eosinophils (CD45^+^, CD11c^-^, SiglecF^+^), *n* = 4. Two-tailed student’s t-test was employed for statistical analysis; Data presented as mean +/− SEM. *< 0.05, **< 0.01, ***< 0.001 and ns= non-significant. Schematic images are sourced by an open-access license from Servier Medical Art

We next investigated whether our findings in mice can be extend to ILC2 function and asthma in humans. A pure population of human ILC2 (hILC2) was FACS-sorted from peripheral blood mononuclear cells (PBMCs) of six healthy donors. The hILC2s were gated as CD45 + , lineage-, CD127^+^, and CRTH2^+^ cells. Following ex vivo culture of hILC2s with vehicle or recombinant human (rh)IL-33, we measured SIRPα expression (Fig. 8A). Confirming our earlier observations in mice, rhIL-33 significantly induced SIRPα and CD47 expression in hILC2s isolated from all six healthy donors (Fig. 8B, C). To assess the clinical relevance of SIRPα, we also evaluated SIRPα expression levels in PBMC-derived ILC2s obtained from asthmatic patients. PBMCs were collected from healthy volunteers and asthma patients in Los Angeles County. Four control subjects were asymptomatic volunteers with no evidence of airflow obstruction, normal lung function, and no history of asthma. Eight asthmatic patients had no asthma exacerbations and had not received oral corticosteroids within three months before study entry. A comparison was made between the SIRPα expression level in hILC2s of asthmatic patients and healthy controls. A notable elevation in SIRPα expression was observed in ILC2s of asthmatic patients in comparison to healthy controls (Fig. S3I). To further investigate the impact of SIRPα stimulation on the activation, proliferation, and function of hILC2s, we cultured cells from each donor with or without CD47-Fc (Fig. 8D). SIRPα stimulation consistently led to a reduction in the nuclear expression of GATA-3 (Fig. 8E) and Ki67 (Fig. 8F), indicative of diminished hILC2 activation and proliferation. Moreover, CD47-Fc treatment consistently reduced the levels of effector cytokines, including IL-4, IL-5, IL-6, and IL-13, in the culture supernatants across all six healthy subjects (Fig. 8G–J). To evaluate the impact of SIRPα on mitochondrial respiration in hILC2s, we measured oxidative metabolism following treatment with CD47-Fc (Fig. 8K). While basal respiration remained comparable between the two groups, SIRPα activation significantly decreased spare respiratory capacity and ATP production rate, indicating a reduction in oxidative energy production (Fig. 8L–N). Furthermore, it was observed that CD47 administration had a significant impact on reducing the development of lung resistance to methacholine in humanized mice, as compared to the vehicle, which supports our previous findings (Fig. 8O–P). Although both groups had the same number of hILC2s originally transplanted, the number of hILC2s recovered in the lungs of CD47-Fc-treated mice on day 4 was lower than in the vehicle group (Fig. 8Q). Finally, the BAL of mice treated with CD47-Fc showed a reduction in hILC2-driven lung inflammation and fewer eosinophils compared to the control group (Fig. 8R). Our findings, consistent with results obtained from mouse ILC2s, underscore the crucial role of SIRPα-CD47 axis in regulating hILC2s. Activated hILC2s exhibited an upregulation of SIRPα, and ex vivo SIRPα stimulation resulted in an overall suppression of hILC2s effector function and oxidative energy production. These results highlight the potential significance of SIRPα-CD47 axis in the modulation of ILC2 responses in the context of human asthma.

## Discussion

Despite the successful application of anti-CD47 and anti-SIRPα blocking antibodies in immunotherapy against cancer cells, the potential of engaging SIRPα to restrain excessive immune activation in the context of ILC2s and allergic lung inflammation remains unexplored. Here, we provide a proof of concept that engagement of SIRPα via its ligand CD47 inhibits ILC2 activation and holds promise for therapeutic intervention in allergic asthma. This study presents novel findings on the crucial function of SIRPα in regulating pulmonary ILC2 proliferation and type 2 cytokine production. The results indicate a significant upregulation of SIRPα following activation of ILC2s by IL-33, both ex vivo and in vivo. Notably, ILC2s with high expression of SIRPα showed increased protein expression of GATA3, Ki67, IL-5, and IL-13 cytokines. We found that SIRPα-deficient mice exhibited exacerbated AHR and lung inflammation, as we confirmed these observations using a SIRPα inhibitor in vivo. To exclude any bystander effects, we measured airway inflammation in alymphoid mice adoptively transferred with SIRPα-deficient or WT ILC2s and found that mice adoptively transferred with SIRPα-deficient ILC2s developed significantly higher lung resistance compared to WT controls. Additionally, we discovered that SIRPα plays a regulatory role in ILC2 activation by controlling mitochondrial function through the regulation of NF-κB via the JAK/STAT, ERK/MAPK, and KLF2 pathways.

The location of SIRPα has been identified as one of the key determinants of receptor activity [23]. Without CD47, SIRPα is relegated to the phosphatase-rich zone outside the immune synapse. This localization not only prevents SIRPα activation but also precludes its interaction with activating receptors. Our study evaluated the effectiveness of exogenous CD47 administration and demonstrated that CD47-Fc engagement of SIRPα, resulting in reduced cytokine secretion and cell proliferative capacity. Although, ILC2s express CD47 and endogenous expression of CD47 in ILC2s can partially limit the ILC2 function, exogenous CD47 can further mitigate the ILC2 proliferation and function, resulting in an improved AHR. These findings suggest that exogenous CD47 may be a potential therapeutic approach in ILC2-mediated AHR, as we demonstrated that CD47-Fc improved AHR in both IL-33 and allergen-induced AHR preclinical models. Additionally, we investigated the effect of CD47 administration on human ILC2s for potential clinical application. Our study demonstrates that the stimulation of SIRPα with CD47-Fc resulted in a reduction of cytokine production, cell proliferation, and mitochondrial function.

SIRPα is an ITIM-containing molecule that effectively governs innate immune activation. Its ubiquitous presence across a spectrum of cells underscores its profound influence on immune homeostasis. Notably, its predominant expression on myeloid immune cells, including neutrophils, monocytes, macrophages, and dendritic cells (DCs), underscores its broad regulatory influence on the immune system [37]. CD47, also known as integrin-associated protein, is the only known endogenous ligand for SIRPα, initiating inhibitory signaling cascades that modulate immune cell functions through diverse mechanisms [38]. Experimental evidence suggests that transient blockade of CD47 and/or SIRPα activates DCs via integrin-mediated mechanisms increases macrophage phagocytic activity and enhances immune cell motility [23, 39, 40]. Recent studies have shed light on the cellular regulatory mechanisms orchestrated by SIRPα in other immune cells. In macrophages, suppression is achieved through the SHP1/2 complex and the regulation of ERK/MAPK signaling pathways, while in DCs, SIRPα modulates cellular functions by inhibiting JAK/STAT signaling [41, 42]. Moreover, the genetic induction of human CD47 on porcine cells demonstrated inhibitory signaling to SIRPα on human macrophages, effectively preventing macrophage-mediated xenograft rejection [43].

Our investigations yield novel insights into the regulatory role of SIRPα in ILC2s, unveiling its involvement in JAK/STAT and ERK/MAPK signaling pathways. Furthermore, we present evidence suggesting the potential regulation of NF-κB by KLF2, highlighting the existence of a cellular regulatory axis mediated by SIRPα in ILC2s [44]. GATA-3 has long been recognized for its critical role in IL-5 and IL-13 secretion by ILC2s [45]. Emerging evidence implicates NF-κB in the regulation of GATA-3 expression in ILC2s [29, 46]. Our findings underscore the substantial impact of modulating SIRPα signaling on GATA-3 expression and the secretion of IL-5 and IL-13 through several key signaling pathways, including JAK/STAT, ERK/MAPK, and KLF2, all of which directly impact NF-κB expression. These insights are further corroborated by observations that SIRPα deficiency leads to increases in STAT3, p38, and p65 subunits. Additionally, stimulation of SIRPα with CD47-Fc results in decreased p65 protein levels, emphasizing the essential role of SIRPα regulation in ensuring proper functioning of ILC2s. Overall, our findings strongly suggest that SIRPα plays a crucial regulatory role in ILC2s through its involvement in multiple signaling pathways that modulate NF-κB expression.

Recent reports suggest that mitochondria play a crucial role in cytokine secretion and cellular functions of ILC2s, opening up promising avenues for further research [32, 47, 48]. ILC2s employ various metabolic pathways, including glycolysis and fatty acid oxidation to fuel the mitochondria and OXPHOS for the production of ATP [49–51]. While investigations have explored the hypothesis that cytokine secretion patterns and cell proliferation may vary depending on the metabolic pathways fueling mitochondrial activity, a definitive consensus remains elusive. Our findings indicate that SIRPα deficiency in ILC2s results in the upregulation of OXPHOS and mitochondrial respiratory gene signatures compared to WT controls. In particular, loss of SIRPα induced the upregulation of TCA cycle enzymes including *Aco2*, *Idh1*, *Idh3a* and *Sucla* which were all recently shown to be controlled by NfKb [29]. Conversely, live oxygen consumption rates were elevated in ILC2s lacking SIRPα, while the mitochondrial activity was further increased in these cells. Next, we further evaluated the impact of SIRPα on mitochondrial respiration in human ILC2s. We assessed oxidative metabolism post-treatment with CD47-Fc, revealing that SIRPα activation markedly decreased spare respiratory capacity and ATP production rates. These findings suggest a notable reduction in oxidative energy production. Thus, our results underscore the crucial regulatory role of SIRPα in modulating ILC2 metabolism and effector functions.

Advancing from murine models to human subjects, our research demonstrates that IL-33 stimulation induces SIRPα expression on human ILC2s (hILC2s). In line with murine data, ex vivo SIRPα activation significantly diminishes the secretion of IL-4, IL-5, IL-6, and IL-13 by ILC2s. Moreover, SIRPα stimulation in hILC2s leads to decreased protein expression of GATA-3 and Ki67, mirroring observations in murine models. Moreover, we evaluated the expression of SIRPα in peripheral blood ILC2 in both healthy subjects and individuals with asthma. Our findings revealed a notable elevation in SIRPα expression in the asthmatic group. While this study had a limited sample size, and further investigation is warranted, these results suggest that SIRPα may serve as a promising therapeutic target for asthma management.

The SIRPα/CD47 signaling axis has emerged as a promising therapeutic target in cancer immunotherapy. Studies have shown that macrophages deficient in SIRPα exhibit heightened cytophagocytic activity and enhanced anti-tumor immunity compared to wild type [17, 18, 52]. Additionally, CD47 expression in cancer cells, such as melanoma and colon cancer, has been linked to immune evasion mechanisms mediated by SIRPα signaling [19, 53]. Moreover, SIRPα/CD47 signaling has garnered attention as a potential therapeutic target in chronic inflammation [17]. SIRPα agonists have demonstrated efficacy in controlling inflammation by suppressing myeloid cell activity in patients with inflammatory bowel disease [37]. This signaling axis has also been implicated in the pathogenesis of type 1 diabetes and graft rejection post-organ transplantation[37, 54, 55]. These findings support our results, confirming the efficacy of targeting SIRPα as a novel inhibitory molecule against allergic lung inflammatory diseases.

Despite advances in effective therapeutics against inflammatory cytokines such as Th2 cytokines and TSLP, a significant number of patients with allergic diseases and asthma do not respond to current therapy, highlighting a substantial unmet medical need. Therapeutic options for targeting innate immune cell infiltrations, such as ILC2s, are limited. Our data suggest that agonizing inhibitory receptor SIRPa, on ILC2s may offer an approach to restrict pathogenic ILC2 infiltration and effector function in the context of allergic airway inflammation.

## Materials and Methods

### Mice

Wild-type (WT) CB57BL/6 J, Rag2^−/−^ (C.B6(Cg)- Rag2tm1.1Cgn/J), Rag2^−/−^ IL-2Rg^−/−^ (Rag2^−/−^GC^−/−^) (C;129S4-Rag2tm1.1Flv Il2rgtm1.1Flv/J), Cd47^−/−^ (CD47 KO) (B6.129S7-Cd47tm1Fpl/J), SIRPα^−/−^ (SIRPα KO) (SIRPα^tm1Ynliu^/J) mice, aged 6-8 weeks, were procured from the Jackson Laboratories (Ann Harbor, ME). 6-8 weeks old age and sex-matched mice were used in the studies. All mice were housed in a pathogen-free animal facility at the Keck School of Medicine, University of Southern California (USC), in accordance with protocols approved by the Institutional Animal Care and Use Committee.

### Murine pulmonary ILC2 isolation and ex vivo culture

To isolate murine pulmonary ILC2s, mice were intranasally challenged with rmIL-33 (BioLegend, 0.5 µg in 40 µL PBS) for three days under anesthesia. On the fourth day, pulmonary ILC2s were sorted using FACS to achieve a purity exceeding 95% on a FACS ARIA III system, as previously described [56, 57]. Following transcranial perfusion with PBS to remove circulating cells, the lungs underwent enzymatic digestion using collagenase Type IV (400 U/mL, Worthington) at 37 °C for 1 h. The resulting suspensions were processed into a single-cell suspension through a 70 µm cell strainer (Falcon). Red blood cells (RBCs) were lysed using RBC lysis buffer (BioLegend) before staining. Live single cells that express CD45, ST2, CD127 and do not express lineage markers (CD3ε, CD4, CD5, TCRβ, TCRγδ, CD45R/B220, CD335, CD11c, CD11b, Gr1, FcεRIα, and Ter119) were considered as ILC2s. This subpopulation was further categorized into SIRPα^-^ and SIRPα^+^ groups based on expressing SIRPα compared to FMO control. Isolated ILC2s were cultured ex vivo at 37 °C in 96-well U-bottom plates at the density of 5 × 10^4^ cell/mL in RPMI medium supplemented with 10% heat-inactivated FBS (Omega Scientific), 100 units/mL penicillin and 100 mg/mL streptomycin (GenClone), rmIL-2 (10 ng/mL, BioLegend), and rmIL-7 (10 ng/mL, BioLegend), henceforth referred to as complete RPMI (cRPMI). For studies involving SIRPα blockade, a monoclonal antibody targeting CD172a (SIRPα) at a concentration of 20 μg/mL (P84, Thermo Fisher) was added to cultures for designated durations. To activate SIRPα/CD47 signaling, the SIRPα ligand, CD47-Fc (Recombinant Mouse CD47-Fc Chimera (carrier-free), Biolegend), was added at a concentration of 20 µg/mL to ILC2 cultures for either 18-24 h or 1 h.

### In vivo murine experiments and BAL collection

Mice were intranasally challenged with either 0.5 µg/mouse rmIL-33 or PBS for three consecutive days under anesthesia, according to established protocols [27]. In experiments involving *A. alternata*, mice received intranasal doses of 100 µg/mouse *A. alternata* for 4 consecutive days under anesthesia, as previously established by our group [28]. Twenty-four hours after the final intranasal challenge, lungs tissues were enzymatically digested, processed into single cell suspensions, and subjected to the designated analyses. For BAL analysis, the airways were lavaged with 1 mL of cold PBS three times and aspirated. BAL samples were then centrifuged to collect cells and the supernatants were preserved for cytokine measurement assays. Prior flow cytometry analysis, cells harvested from BAL samples underwent treatment with RBC lysis buffer for optimal staining.

### Flow cytometry

The following panel of murine antibodies were utilized: FITC anti-mouse lineage markers [**CD3ε** (145-2C11), **CD4** (GK1.5), **CD5** (53-7.3), **TCRβ** (H57-597), **TCRγδ** (UC7-13D5), **B220/CD45R** (RA3-6B2), **Gr-1** (RB6-8C5), **CD11c** (N418), **CD11b** (M1/70), **Ter119** (TER-119), **FcεRIα** (MAR-1), **CD335** (29A1.4)], PE-Cy7 anti-mouse **CD127** (A7R34), APC-Cy7 anti-mouse **CD45** (30-F11), PE-Cy7 anti-mouse **CD45** (30-F11), Brilliant Violet 510™ anti-mouse **CD45.1** (A20), Alexa Fluor®488 anti-mouse **CD45.2** (104), APC-Cy7 anti-mouse **CD11c** (N418), FITC anti-mouse **CD19** (6D5), APC anti-mouse **Gr-1** (RB6-8C5), PerCP-Cy5.5 anti-mouse **CD3e** (17A2), Brilliant Violet 510™ anti-mouse **CD172a (SIRPα)** (P84), and Brilliant Violet 421™ anti-mouse **CD47** (miap301), all acquired from Biolegend. Additionally, PE anti-mouse **SiglecF** (E50-2440) was purchased from BD Biosciences, while PerCP-eFluor710 anti-mouse **ST2** (RMST2-2) and eFluor450 anti-mouse **CD11b** (M1/70) were obtained from Thermofisher. When indicated, **MitoTracker™ Green FM Dye**, for flow cytometry (Thermofisher) was employed as per manufacturer instructions. Intracellular staining was performed using the BD Cytofix/Cytoperm kit following the manufacturer’s instructions. Cells were stimulated 4 h ex vivo with 50 µg/mL PMA, 500 µg/mL ionomycin (both from Sigma) and 1 µg/mL Golgi plug (BD Biosciences), PE anti-mouse **IL-13** (eBio13A, Thermofisher), and APC anti-mouse **IL-5** (TRFK3, biolegend) were used for intracellular cytokine staining.

For intranuclear staining, the Foxp3 Transcription Factor Staining Kit (Thermofisher) was used along with APC anti-mouse **Ki67** (SolA15, Thermofisher), and PE anti-mouse/human **GATA-3** (TWAJ, Thermofisher). Phosphorylation assessment involved FITC anti-human/mouse Phospho **STAT3** (RUO, Biosciences), PE anti-human/mouse **Rela** (p65) (532301, R&D systems), APC anti-human/mouse Phospho **p38** (4NIT4KK, Invitrogen), and Alexa Fluor™ 647 anti-human/mouse **KLF2** (bs-2772R, Bioss). Apoptosis staining was performed using PE **Annexin V** (Thermofisher) and **DAPI** (Sigma), based on the manufacturer instructions.

The following human antibodies were utilized: FITC anti-human lineage cocktail including **CD3** (UCHT1), **CD14** (HCD14), **CD16** (3G8), **CD19** (HIB19), **CD20** (2H7), **CD56** (HCD56). Additional lineage markers encompassing FITC anti-human **CD235a** (HI264), FITC anti-human **FCεRIα** (AER-37), FITC anti-human **CD1a** (HI149), FITC anti-human **CD123** (6H6) and FITC anti-human **CD5** (L17F12) we also added to the cocktail. APC-Cy7 anti-human **CD45** (HI30), PE-Cy7 anti-human **CD127** (A019D5), and PE anti-human **CRTH2** (BM16) were all purchased from Biolegend, while eFluor450 anti-human **Ki-67** (20Raj1) was obtained from Thermofisher. BD OptiBuild™ BV421 Mouse Anti-Human **CD172a/b (SIRPα)** was purchased from Bioscience**. Live/dead fixable** violet or aqua cell stain kits (Thermofisher) were used to exclude nonviable cells, and **CountBright absolute counting beads** (Thermofisher) were applied for absolute cell number calculations. Stained cells were analyzed on a FACSCanto II system, and the data was analyzed using FlowJo version 10 software.

### Lung function and histology assessment

Lung function was evauated using the FinePointe RC system (Buxco Research Systems, Wilmington, NC). Mice were anesthetized and mechanically ventilated according to established protocols to ensure their comfort and well-being dring the assessment [58]. Aerosolized PBS (baseline) and escalating doses of methacholine (Sigma) ranging from 5 to 80 mg/mL were administered to the mice. Maximal pulmonary resistance (R_L_) and minimal dynamic compliance (cDyn) were recorded during a 3 min interval following each challenge. For histologic analysis, the right lung lobe was excised and preserved in 10% PFA. The lungs were then embedded in paraffin, and 4 mm sections were prepared for subsequent hematoxylin and eosin (H&E) staining. Composite figures were generated from the resulting images using Adobe Illustrator software (version 22.1). Histological samples were visualized using a KeyenceBZ-9000 microscope (Keyence, Itasca, Ill) and analyzed with ImageJ Analysis Application (NIH & LOCI, University of Wisconsin).

### Assessment of mitochondrial function

The real-time oxygen consumption rate (OCR) was determined using the Seahorse Mini HS XF instrument (Agilent). Briefly, 6 × 10^4^ ILC2s were treated with or without human CD47-Fc for a duration of 24 h. Subsequently, the cells were enumerated and transferred into a Seahorse XFp PDL-coated cell culture miniplate in triplicates. ILC2s were cultured in FBS/Phenol red-free Seahorse media supplemented with 10 mM glucose, 2 mM glutamine, and 1 mM pyruvate. Then, the assay designed to profile the metabolic activity of T-cells (Agilent) was employed. Baseline measurements were established, followed by sequential injections of 1.5 µM oligomycin, 2.5 µM BAM15, and 0.5 µM rotenone/antimycin A into the culture. Oxygen levels in the culture were measured in triplicate following each injection. Parameters including basal respiration, spare respiratory capacity, glycoATP rates (ATP produced from metabolizing glucose to lactate), mitoATP rates (ATP produced during OXPHOS), and total ATP rates (glycoATP + mitoATP) were computed. The OCR data were normalized to the average count of ILC2s per well. For the purpose of conducting experiments to inhibit mitochondrial activity, 1 × 10^4^ ILC2 cells were selected from SIRPα KO or CD47 KO mice that has been stimulated with IL-33 and had been cultured with the mitochondrial complex I inhibitor BAY 87-2243 (28626), purchased from Cayman, at a concentration of 10 µM for a period of 24 h.

### Analysis of transcriptomic profiling data

ILC2 were harvested and lysed in RLT buffer (Qiagen), followed by RNA extraction utilizing the MicroRNeasy kit (Qiagen). For cDNA synthesis, 10 pg of RNA from each sample was utilized as input with the SMARTer Ultra Low Input RNA v3 kit (Clontech) for library preparation. Following sample amplification, sequencing was performed on a NextSeq 500 system (Illumina), yielding an average of approximately 30 million reads per sample.

The raw reads underwent processing utilizing Partek Genomics Suite software, version 7.0 (Partek Inc). Subsequently, alignment of the raw reads was accomplished using STAR – 2.6.1d with the mouse reference index mm10 and GENECODE M21 annotations. Following alignment, further quantification and normalization of the reads were conducted, and differential analysis was executed employing DESeq. Gene enrichment analysis was subsequently performed utilizing the Ingenuity Pathway Analysis (IPA) tool. Additionally, for the analysis of single-cell RNA sequencing (scRNA-seq) data, the raw files were retrieved and reanalyzed according to methods detailed in a previous study [59]. Publicly available datasets were downloaded [25, 26] for the analysis of *Sirpa* and *Cd47* expressions. A variety of R packages were utilized for the creation of graphical representations and data visualizations. These included the circlize package (version 0.4.15) for chord diagrams and the ggplot2 package (version 3.4.3) for the customization and generation of clear, data-driven visualizations. The raw and processed data have been uploaded to the Gene Expression Omnibus database (GSE264061).

### Quantification of cytokines

Following treatment of ILC2s with indicated reagents, culture supernatants were obtained for subsequent analysis. Utilizing the LEGENDplex™ Mouse Th2 Panel (Biolegend), cytokine levels were assessed in accordance with the manufacturer’s instructions. Similarly, cytokine measurements from BAL fluid were conducted by harvesting associated supernatants using identical procedures. For human cytokine analysis, culture supernatants were gathered, and cytokine levels were evaluated utilizing the LEGENDplex™ Human Th2 Panel (Biolegend).

### Culture and Isolation of human ILC2

Human peripheral ILC2s were isolated with high purity ( > 95%) from PBMCs of 6 healthy donors utilizing the FACSARIA III system. Fresh human blood was initially diluted at a 1:1 ration in PBS, followed by PBMC isolation using SepMateTM-50 separation tubes (STEMCELL Technologies) according to the manufacturer’s instructions. Subsequent to RBC lysis (Biolegend), CRTH2^+^ cells were isolated using the CRTH2 MicroBead Kit (Myltenyi Biotec) in accordance with the manufacturer’s protocol. Human ILC2s were phenotypically characterized as CD45^+^, CD127^+^ and CRTH2^+^ cells negative for lineage markers (CD3, CD5, CD14, CD16, CD19, CD20, CD56, CD235a, CD1a, CD123). These isolated ILC2s were then cultured for 72 h at 37 °C (5 × 10^4^/mL) in cRPMI supplemented with rhIL-2 (10 ng/mL, Biolegend) and rhIL-7 (10 ng/mL, Biolegend) with or without rhIL-33 (100 ng/mL, Biolegend) in U-bottom 96-well plates. For designated analyses, 20 µg/mL of human CD47-Fc (4670-CD, R&D) was added to the cultures. In experiments involving humanized mice, the human ILC2s were cultured with rhIL-2, rhIL-7, and rhIL-33 for a period of 14 days. The culture medium was changed every 2-3 days until the desired number of ILC2s was achieved. Following this, a total of 2 × 10^5^ ILC2s were transferred intravenously to Rag2^−/−^GC^−/−^ mice, alymphoid hosts. Mice were subjected to a series of challenges with carrier-free rhIL-33 (BioLegend) diluted in 50 μl PBS at a concentration of 1.0 μg/mouse for 3 consecutive days while under anesthesia, as previously described [44]. Before each intranasal challenge, CD47-Fc (4670-CD, R&D) was intravenously administered at a dose of 50 μg/mice in 200 μl PBS.

#### Human cohorts of patients with asthma

All study patients and healthy controls underwent a blood draw. A total of 5–10 mL of whole blood was collected in EDTA-treated tubes and processed rapidly to obtain a single-cell suspension for the presence of lineage-, CD127^+^, and CRTH2^+^ ILC2s, as previously described [60]. The expression of SIRPa was quantified as mean fluorescence intensity (MFI) with each sample stained with its own SIRPa staining control for normalization. To determine the absolute cell numbers and the number of ILC2s per 10 mL of blood, CountBright absolute counting beads (Thermofisher) were employed.

### Statistical analysis

Data are presented as mean ± SEM and analyzed utilizing GraphPad Prism software (version 9.5.1). Two-tailed Student’s t-tests for unpaired data were employed for comparing two groups, while one-way analysis of variance (ANOVA) tests was utilized for multi-group comparisons.

## Supplementary information

Supplementary figure 1-3

Supplementary figure 1-3 Legend

## Data Availability

The analysis in this paper utilized pre-existing publicly available scRNA-seq data (CRA004586 and GSE264408). The bulk RNA-seq data have been uploaded to the Gene Expression Omnibus database (GSE264061). Additional information necessary to facilitate the reanalysis of the data featured in this paper will be made available by the lead contact upon reasonable request.
