# Supplementary figures and images for: SIRPα engagement regulates ILC2 effector function and alleviates airway hyperreactivity via modulating energy metabolism
Source: Cell Mol Immunol. 2024 Aug 19;21(10):1158–74. doi: 10.1038/s41423-024-01208-z (PMC11442993; doi:10.1038/s41423-024-01208-z)

A

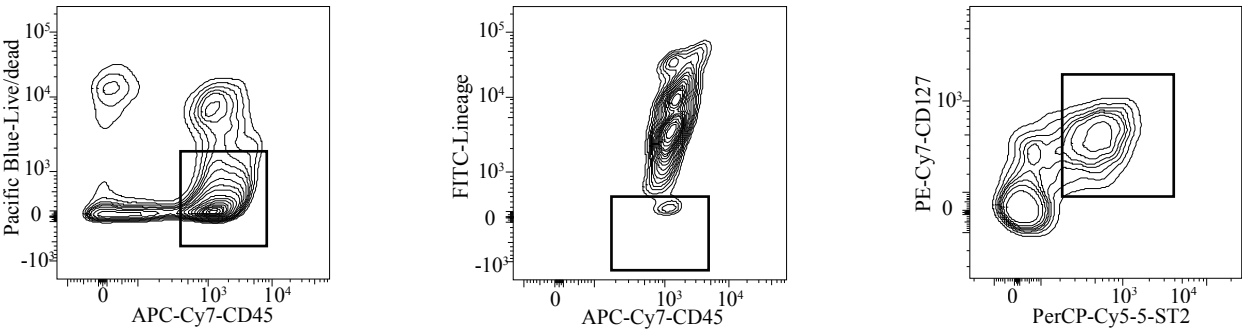

B

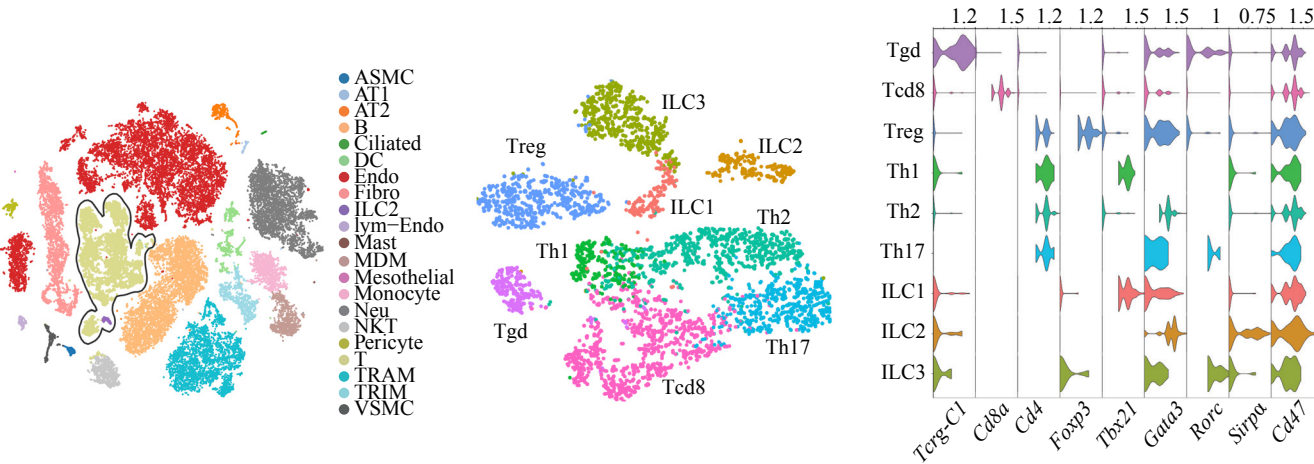

C

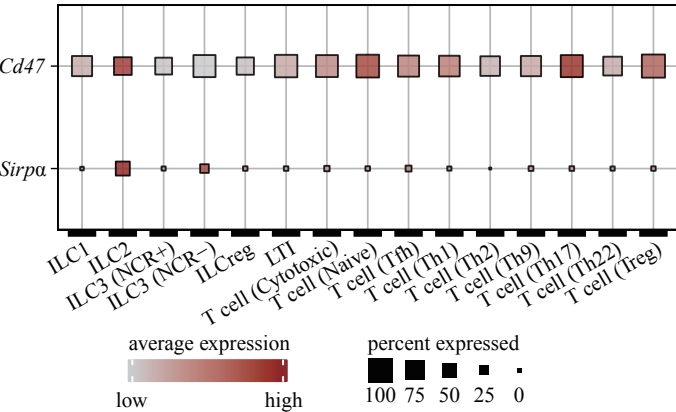

D

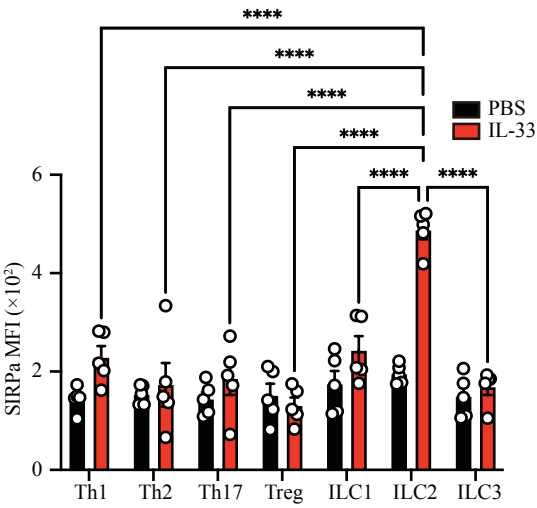

E

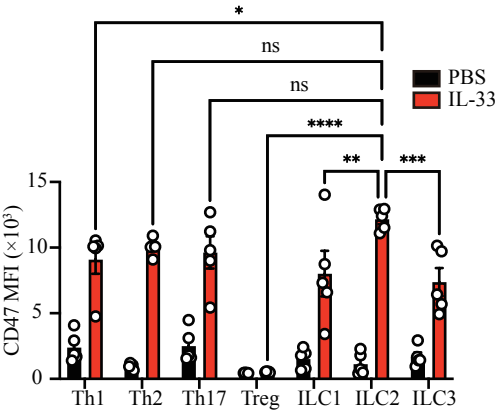

F

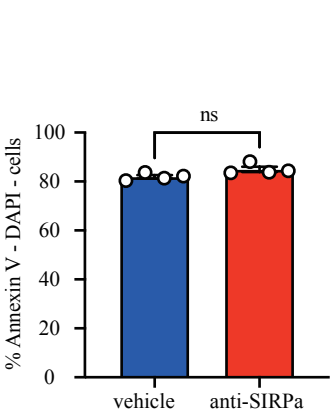

G

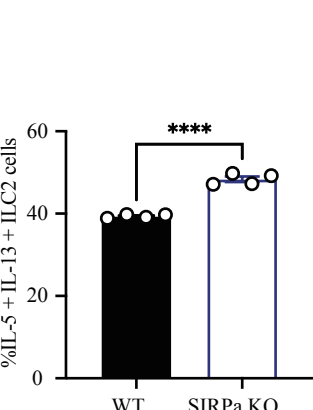

Supplementary Figure. 2

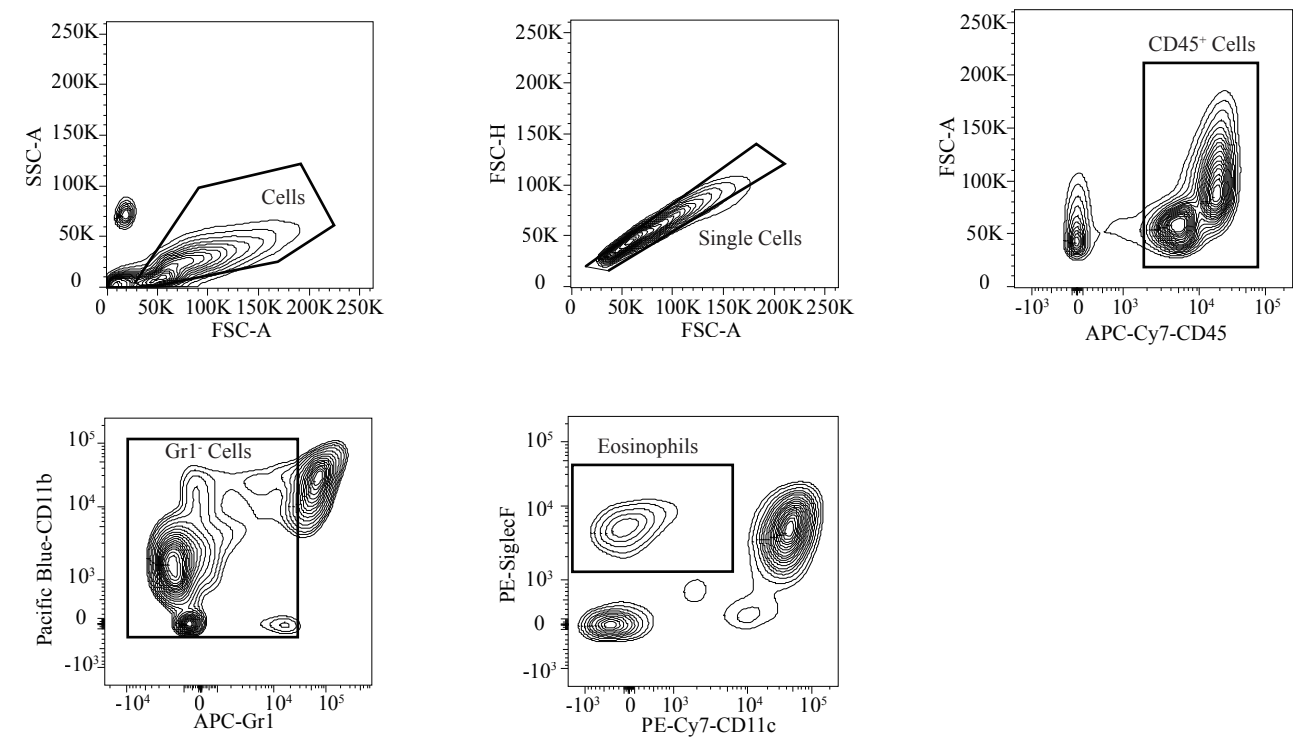

Supplementary Figure. 3

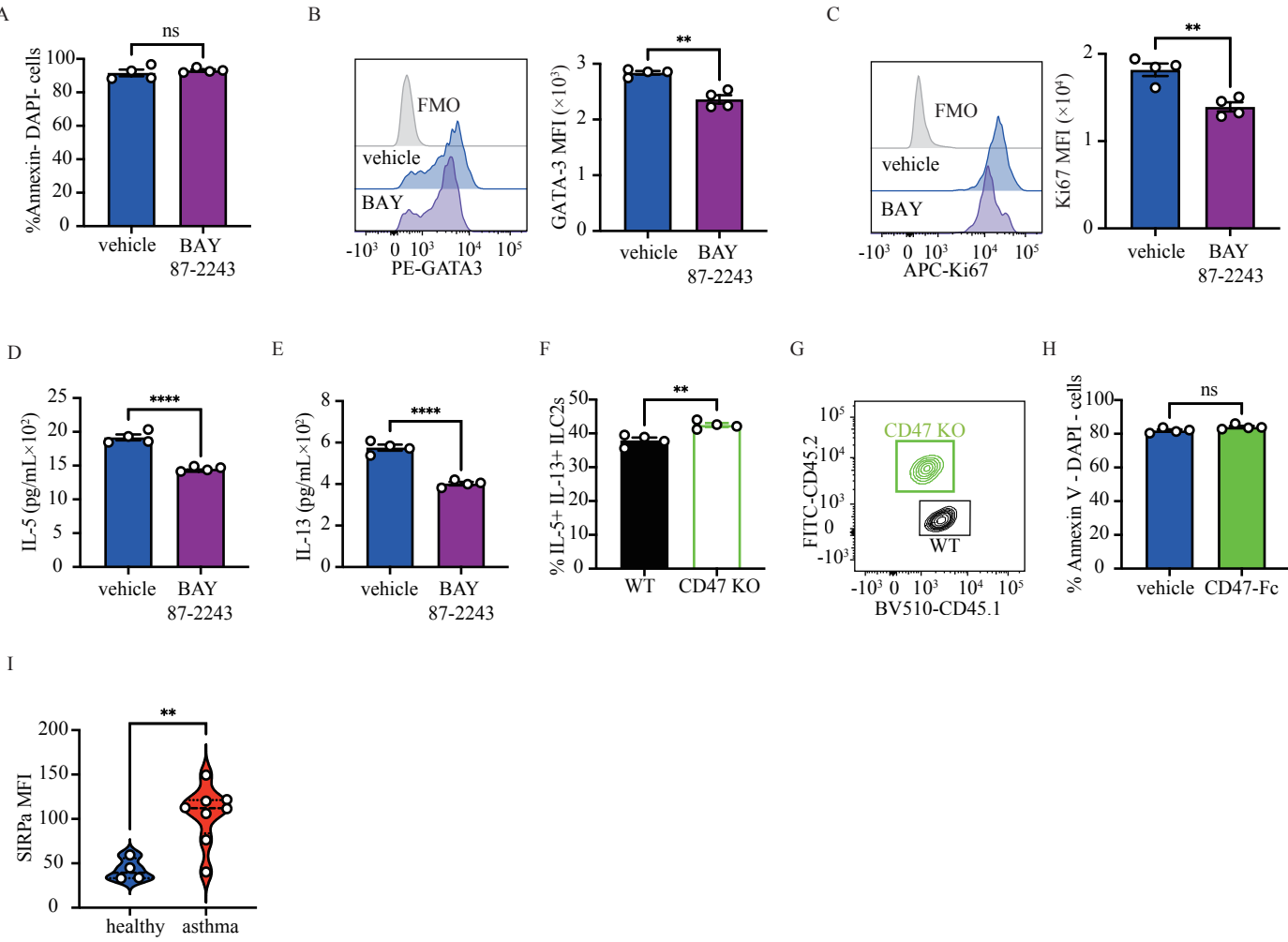

Supplement: Supplementary file 1 — Supplementary figure 1-3 [file 41423_2024_1208_MOESM1_ESM.pdf]
