# Supplementary material for: SIRPα engagement regulates ILC2 effector function and alleviates airway hyperreactivity via modulating energy metabolism
Source: Cell Mol Immunol. 2024 Aug 19;21(10):1158–74. doi: 10.1038/s41423-024-01208-z (PMC11442993; doi:10.1038/s41423-024-01208-z)
Supplement: Supplementary file 2 — Supplementary figure 1-3 Legend [file 41423_2024_1208_MOESM2_ESM.docx]

**Figure S1. Gating strategies, measuring expression, and cytotoxicity assay.**

**(A**) Lung ILC2 gating strategy, defined as CD45^+,^ Lineage^-^, CD127^+^, ST2^+^ cells. **(B)** scRNA-seq was downloaded from the mouse Th2 asthma model public database (CRA004586) and analyzed the expression level of *Sirpα* and *Cd47*. The left panel showed that t-SNE plot revealing on main immune cell populations annotated in left panel. A more comprehensive examination of the T cell subsets permitted the classification of these cells into the categories depicted in the middle panel. The figure on the right panel displays the expression of *Sirpα* and *Cd47* across various subsets of immune cells, including T helper (Th1, Th2, Th17), regulatory T cells (Treg), innate lymphoid cells (ILC1, ILC2, and ILC3). **(C)** scRNA-seq was downloaded from Inflammatory bowel disease mouse public database (GSE264408) and analyzed the expression level of *Sirpα* and *Cd47*.

**(D)** The quantification of SIRPα expression in immune cells was presented as MFI; n=4. **(E)** The quantification of CD47 expression in immune cells was presented as MFI; n=4. **(F)** Annexin-V/DAPI assay following treatment with anti-SIRPα antibody; n=3. Frequency of double negative (live) cells are presented as bar charts. **(G)** Frequency (%) of IL-5^+^ and IL-13^+^ ILC2s in both WT and SIRPα KO mice; n=4. Two-tailed student’s t-test was employed for statistical analysis; Data presented as mean +/- SEM. *<0.05, **<0.01, ***<0.001, ****<0.0001, and ns= non-significant.

**Figure S2. BAL gating strategy.**

Leukocytes are gated as CD45^+^ cells, and eosinophils are defined as CD45^+^, Gr1^-^, CD11c^-^, and SiglecF^+^ cells.

**Figure S3. The inhibition of mitochondrial function and measuring the expression.**

**(A)** Annexin-V/DAPI assay following treatment with BAY 87-2243; n=4. Frequency of double negative (live) cells are presented as bar charts. **(B, C)** Expression of GATA-3 **(B)** and Ki67 **(C)** in vehicle and BAY 87-2243 groups is depicted. Corresponding quantitation is presented as MFI; n=4. **(D, E)** Levels of IL-5 **(D)** and IL-13 **(E)** production in the culture supernatant were measured; n=4. **(F)** Frequency (%) of IL-5^+^ and IL-13^+^ ILC2s in both WT and CD47 KO mice; n=4. **(G)** Co-culture experiment gating. **(H)** Annexin-V/DAPI assay following treatment with CD47-Fc; n=3. Frequency of double negative (live) cells are presented as bar charts. **(I)** Expression level of SIRPα on peripheral hILC2s, presented as MFI. Two-tailed student’s t-test was employed for statistical analysis; Data presented as mean +/- SEM. *<0.05, **<0.01, ****<0.0001, and ns= non-significant.
